# Series of Near-IR-Absorbing Transition Metal Complexes with Redox Active Ligands

**DOI:** 10.3390/molecules25112531

**Published:** 2020-05-29

**Authors:** Esko Salojärvi, Anssi Peuronen, Manu Lahtinen, Hannu Huhtinen, Leonid S. Vlasenko, Mika Lastusaari, Ari Lehtonen

**Affiliations:** 1 Inorganic Materials Chemistry research group, Department of Chemistry, University of Turku, FI-20014 Turku, Finland; emsalo@utu.fi (E.S.); anssi.peuronen@utu.fi (A.P.); miklas@utu.fi (M.L.); 2 Department of Chemistry, P.O. Box 35, University of Jyvaskyla, FI-40014 Jyvaskyla, Finland; manu.k.lahtinen@jyu.fi; 3 Wihuri Physical Laboratory, Department of Physics and Astronomy, University of Turku, FI-20014 Turku, Finland; hannu.huhtinen@utu.fi (H.H.); leonidvlasenko@yahoo.com (L.S.V.); 4 Ioffe Institute, Russian Academy of Sciences, 194021 St. Petersburg, Russia

**Keywords:** metal organic complex, redox-active ligand, non-innocent ligands

## Abstract

New soluble and intensely near-IR-absorbing transition metal (Ti, Zr, V, Ni) complexes were synthesized using a redox non-innocent *N*,*N*’-bis(3,5-di-tertbutyl-2-hydroxy-phenyl) -1,2-phenylenediamine (H_4_L) as a ligand precursor. In all the complexes, ([Ti(L^ox^)_2_, [Zr(L^ox^)_2_], [V(L^sq1^)(HL^ox^)] and [Ni(HL^ox^)_2_], two organic molecules coordinate to the metal center as tri- or tetradentate ligands. The solid-state structures of the complexes were determined using single crystal XRD, and the compounds were further characterized with Electrospray Ionisation Mass Spectrometry (ESI-MS). Thermoanalytical measurements indicated the thermal stabilities of the complexes. All compounds absorb strongly in the near-IR region and show very interesting magnetic and electrochemical properties. Moreover, it was shown that the V and Ni complexes can also convert absorbed near-IR photons to (un)paired electrons, which indicates great promise in photovoltaic applications.

## 1. Introduction

**Scheme 1:**
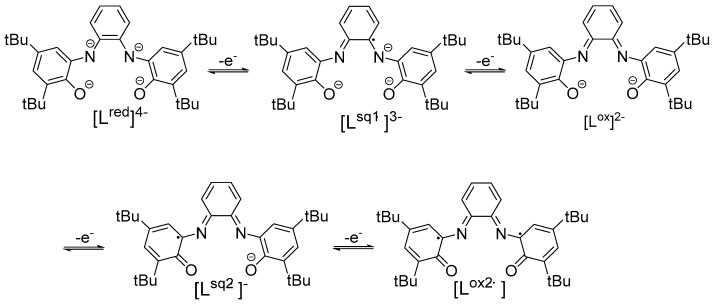
The different oxidation states of the deprotonated ligand L (adapted from the reference [8]).

In coordination chemistry, a ligand is referred as ‘non-innocent’ when it forms complexes where the oxidation state of the central atom cannot be absolutely defined [1]. For example, o-aminophenol *N*,*N*′-bis(3,5-di-tertbutyl-2-hydroxy-phenyl)-1,2-phenylenediamine H_4_L (the oxidation states of L, Scheme 1) has a rich redox-chemistry and its complexing abilities towards transition metals, i.e., Ti, Zr, Hf, Mo, W, Mn, Co, Cu and Zn, have attracted substantial interest over the past few years [2,3,4,5,6,7,8]. Earlier studies show that this proligand can form complexes with various transition metals in 1:1 and 2:1 stoichiometry. Moreover, the galore electrochemical behavior of H_4_L (from hereon L corresponds to the ligand in any oxidation state) allows five different oxidation states (Scheme 1). The foremost motivation for research on such redox-active ligands has been in the development of new homogenous catalysts, which could utilize these ligands as electron reservoirs during the catalytic cycle. On the other hand, the redox activity of the ligand may allow the corresponding metal complexes to have multiple low-energy charge transfer processes within, thus rendering the complex able to absorb electromagnetic radiation at the visible and the near-IR regions intensely.

There are many metal-organic transition metal complexes with redox-active ligands which are studied for their potential applicability for strongly near-infrared (NIR)-absorbing dyes [9,10,11,12,13,14,15,16,17,18,19] or as single molecule magnets [20,21,22,23] (SMMs). NIR absorption is important for dye-sensitized solar cells [20,21,22,23], camouflaging, optical NIR filtering and for NIR photodetectors. Metal organic NIR dyes are also studied for their high thermal durability and lightfastness compared with organic NIR dyes [24]. As H_4_L is a redox-active ligand that can have, in certain protonation states, delocalized π-conjugated systems, it can form stable radicals upon complexation with transition metal ions [8]. Stable radicals, i.e., compounds carrying unpaired electrons, may have strong interactions with photons and therefore exhibit strong absorption at UV-Vis-NIR range. In this study, we have used H_4_L as a ligand with the aim of preparing new NIR-absorbing transition metal complexes and have studied their molecular structures as well as their magnetic properties.

## 2. Results and Discussion

### 2.1. Synthesis of Complexes

**Scheme 2:**
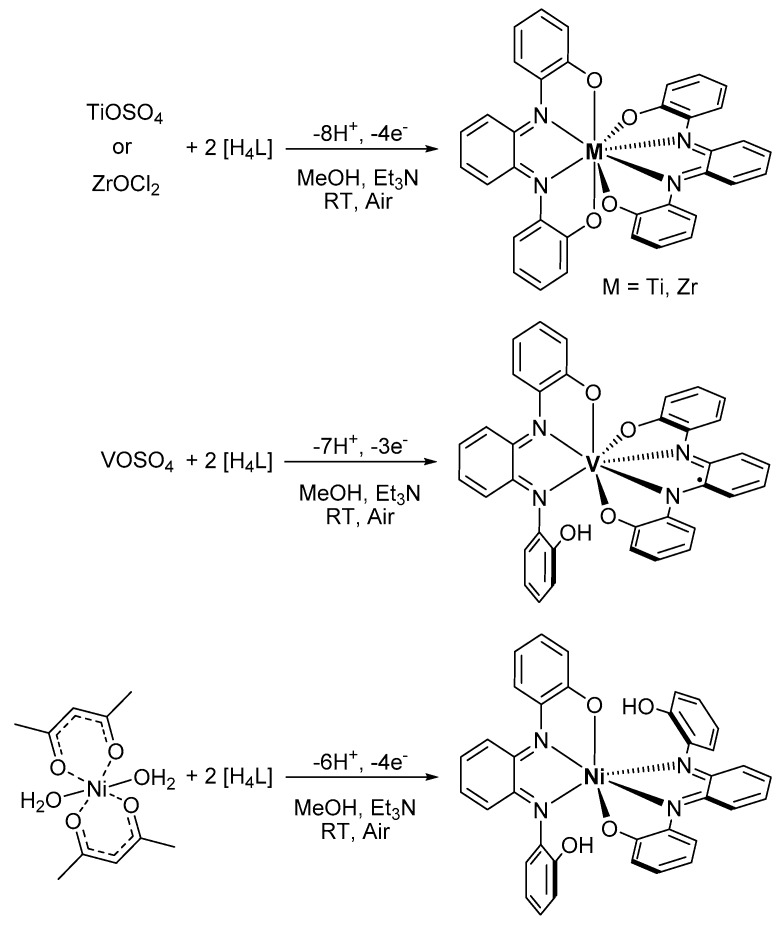
The syntheses of the complexes. The *tert*-butyl groups are omitted for clarity.

The reaction between metal precursors and H_4_L in basic methanol solutions under ambient atmosphere afforded the dark crystals of mononuclear complexes (Scheme 2). All the reactions yielded mononuclear complexes with a 2:1 ligand-to-metal ratio regardless of the equivalent ratios of the starting compounds. The complexes were isolated as dark crystals either directly from the reaction mixture or with subsequent crystallization. The synthesis of [V(L^sq1^)(HL^ox^)] was repeated using vanadyl acetylacetonate as a metal precursor to yield a chemically identical product, which crystallized in an orthorhombic crystal system. No further analyses were run utilizing the orthorhombic form. Powder X-ray diffraction (PXRD) was used to establish that the material used for all analyses corresponds to the monoclinic (*C*2/*c*) structure (see Supplementary Materials for PXRD analysis and the crystal structure of the orthorhombic polymorph). All complexes are air- and moisture-stable at room temperature. In the DSC-TGA (Differential Scanning Calorimetry-ThermoGravimetric Analysis) measurements, which were carried out with a heating rate of 5 K/min for all complexes in nitrogen atmosphere, it was found that the Ti complex loses ca. 8% of the sample’s weight, which could be attributed to the loss of solvate molecules from the crystal lattice (XRD measurements, see below). The weight then remains stable until ca. 270 °C, where it starts to rapidly decline, probably due to the decomposition of the molecule. The other complexes were found to be stable up to 340 °C, 250 °C and 230 °C, for Zr, V and Ni complexes, respectively, until decomposition occurred. 

### 2.2. Structural Studies

**Figure 1:**
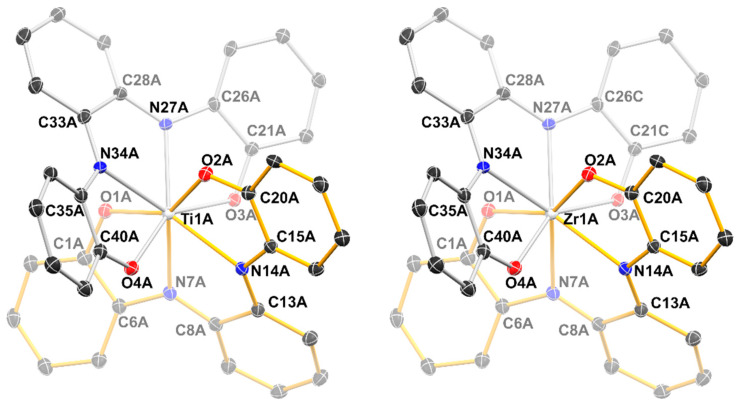
The crystal structures of [Ti(L^ox^)_2_] and [Zr(L^ox^)_2_]. The hydrogen atoms and *tert*-butyl groups are omitted for clarity. Thermal ellipsoids are drawn at the 30% probability level.

The single crystal X-ray diffraction studies (see Supplementary Materials, Table S1) showed that both Group IV complexes crystallize in a triclinic unit cell with two distinct complex molecules in the asymmetric unit. Ti complex crystallizes as an acetonitrile solvate, whereas Zr complex has a large, ca. 1000 Å^3^, void space in the crystal lattice with unresolved electron density. This void could potentially be large enough to accommodate an additional uncoordinated ligand. This is, however, not supported by ^1^H-NMR spectroscopic evidence which shows a methanol solvent peak instead. Therefore, the electron density in the void was treated as solvent using Olex^2^ solvent mask feature. In both cases, the complexes are formed of neutral molecules in which two nearly identical organic ligands are fully deprotonated (N-H and O-H protons) and coordinated to the metal center as tetradentate ONNO-donors. The coordination geometry around the central atom is best described as a distorted square antiprism with phenolic oxygens coordinated to the central atom in a *mer* form. The C-N and C-C interatomic distances in the central phenylene rings of each distinct ligand suggest a localized, i.e., a ‘cyclohexadiene diimine’-like, structure rather than a delocalized system (bond parameters are presented in Table 1). The four Ti-N bonds (Table 1) display significant elongation compared to the previously published [Ti(L^red^)(py)_2_] complex but are very similar to the [Ti(L^ox^)(Cl)_2_] complex [4]. Although the long Ti-N distances can be caused by ligand-ligand steric effects, the discussed interatomic distances strongly suggest that the ligands in the presented [Ti(L)_2_] complex adopt the [L^ox^]^2−^ form. Furthermore, according to Brown, it is possible to use the metrical oxidation state (MOS) to estimate the formal oxidation states of the metal-coordinated *o*-aminophenol moieties based on their geometrical parameters, i.e., the bond lengths of O-C and N-N bonds and the C-C bonds of the phenyl ring [25] or [Ti(L)_2_], these calculations gave the MOS values of −1.09(11), −1.22(6), −1.22(9) and −1.37(8), which support the idea that both ligands adopt the partially oxidized [L^ox^]^2−^ form. The overall structure of the [Zr(L)_2_] complex is very similar to that of its Ti analogue (Figure 1), and the bonding parameters (Table 1) likewise indicate that the organic ligands adopt the [L^ox^]^2−^ oxidation state. Hence, both complexes can be described as [M(L^ox^)_2_], although the MOS values of the *o*-aminophenol moieties in [Zr(L^ox^)_2_] are slightly higher [−1.21(14), −1.39(18), −1.40(14) and −1.41(12)] compared to [Ti(L^ox^)_2_]. However, it is known that complexes of high oxidation state metals may have strong π-donation from the high-lying π orbitals of amidophenoxide ligands, which may cause the metrical oxidation state to differ from the theoretical value [25]. It therefore seems obvious that the formal oxidation state of both Ti and Zr centres should be assigned as +4.

According to the single crystal XRD analysis, the vanadium complex is formally a neutral species, where two ligands show different coordination modes and protonation states, i.e., [V(L)(HL)]. One of the two distinct ligands is coordinated to the central metal ion as a fully deprotonated tetradentate ONNO-donor, whereas the second ligand is coordinated as a partially deprotonated tridentate ONN-donor. The sole phenolic hydroxyl group that remains protonated and uncoordinated forms a hydrogen bond to the phenolate oxygen of the four-dentate ligand [*d*(O1A···O4A = 2.967 Å)]. The phenolic oxygens are aligned in a *mer*-fashion and the geometry of the central V atom is best described as a distorted pentagonal bipyramid with the tridentate ligand in a planar geometry (dihedral V-N-N-O angle is −4.6°) and the tetradentate clearly twisted (dihedral O-N-N-O angle is 37.0°). Interestingly, the crystal packing of [V(L)(HL)] is isostructural with the known Mo^6+^ and W^6+^ complexes which have the formula [M(L)^red^(HL)^sq1^] [5,6], even if the formal oxidation state in the vanadium complex is apparently lower.

**Figure 2:**
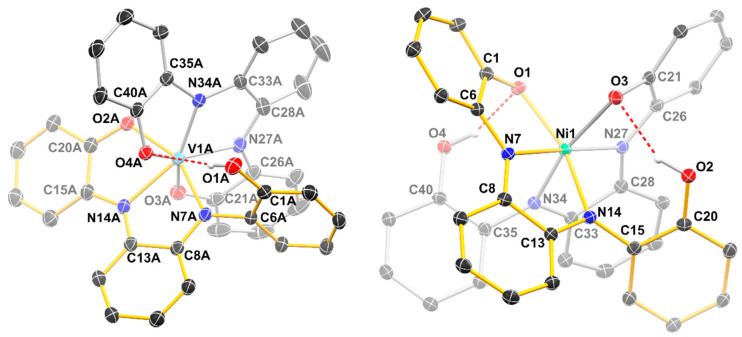
The crystal structures of [V(L^sq1^)(HL^ox^)] and [Ni(HL^ox^)_2_] with the intramolecular hydrogen bonds drawn with dashed lines. The C–H hydrogen atoms and *tert*-butyl groups are omitted for clarity. Thermal ellipsoids are drawn at the 30% probability level.

Like the isostructural Mo and W complexes, [V(L)(HL)] consists of two distinct molecular units in the asymmetric unit. The intramolecular bond parameters between these units are very similar and thus the complex V1A is taken as an example while discussing the bonding characteristics of the complex. The intramolecular distances are shown in (Table 2, Figure 2). The C-C bond distances in the central six-membered C_6_ rings indicate that the tridentate ligand bears a cyclohexadiene backbone whereas the tetradentate ligand shows a more delocalized C_6_ system (Table 2). This is also reflected in the C-N bond distances, which for the tridentate ligand are ca. 0.05–0.07 Å shorter compared with the tetradentate ligand and are closer to values that would be expected for an imine. MOS calculations for the two distinct *o*-aminophenols of the tetradentate ONNO ligand gave the values of −1.65(12) and −1.79(11), thus yielding an approximate formal oxidation state of −3 for the entire ligand. The fully coordinated *o*-aminophenol moiety of the trisdentate ONN ligand, on the other hand, yields a MOS value of −1.10(6). This information, combined with the magnetic measurements (vide infra), indicates that the complex is expected to have one tetradentate and triply anionic [L^sq1^]^3−^ ligand, with an unpaired electron, as well as one trisdentate, partly protonated and singly anionic [HL^ox^]^−^ ligand and V^4+^ *d*^1^ central metal ion. Hence, although [V(L^sq1^)(HL^ox^)] shows remarkable structural resemblance to its formerly synthesized Mo [7] and W [6] analogues, the oxidation states of both chelating ligands are different to the group VI metal complexes reported earlier. This reveals that the overall structure of the ligand is not entirely dependent on the oxidation state.

[Ni(HL)_2_] is formed of neutral molecules, where the two organic ligands are coordinated to the metal centre as three-dentate ONN-donors in a nearly symmetric fashion. In both ligands, the shortest C–N bonds (1.308, 1.310, 1.323 and 1.335 Å) are towards the central phenylene ring of the ligand corresponding to imine double bonds (see Table 2). In general, the bonding parameters indicate that the organic ligands are in a partly deprotonated [HL^ox^]^−^ oxidation state with each ligand having one dangling phenolic hydroxyl group which engages in intramolecular hydrogen bonding with the coordinated O-atoms. These two coordinated phenolic oxygens (O1 and O3) are aligned in a *cis* form in respect to the central atom. The coordination geometry around the central atom is best described as a distorted octahedron, which matches with the proposed structure for the high-spin Ni^2+^ complex. The calculated MOS values are −0.92(14) and −1.15(12) for the coordinated o-aminophenol moieties. These findings support strongly the idea of two identical [L^ox^]^2−^ ligands within the complex.

### 2.3. NMR and MS Spectroscopic Studies

The ^1^H-NMR spectra of Ti and Zr complexes display two distinct singlets (*ca*. 1.2 and 0.9 ppm) for the *tert*-butyl groups and four multiplets (ca. 6.95–7.55 ppm) for aromatic protons demonstrating the high symmetry of the molecules in the solutions. Although the ligand H_4_L can form stable radicals upon coordination and intramolecular redox reactions, the well-resolved NMR spectra (see Supplementary Information) verify the diamagnetic nature of the Ti and Zr complexes at room temperature in solution. The protonation states of the coordinated ligands were also studied using electrospray ionization (ESI-MS) mass spectroscopy, and the molecular ions [M + H]^+^ were detected at *m*/*z* = 1073.62 for [Ti(L^ox^)_2_] and at *m*/*z* = 1115.62 for [Zr(L^ox^)_2_], which indicates that the ligands are fully deprotonated upon coordination.

The NMR studies for the V and Ni complexes provided little information on the molecular structure in solution, indicating the paramagnetic nature of the complex. Therefore, the Evans NMR method [26] for measuring the magnetic susceptibilities in solutions was applied to get µ_eff_ = 0.66 µ_B_ for the V complex. This effectively equals to less than half spins on the molecule, thus suggesting that the electronic ground state of the complex is a mixture of singlet and triplet/pentet states, which is also supported by SQUID (Superconducting Quantum Interference Device) results (see below). Although we cannot entirely rule out the possibility of paramagnetic impurity being the source of the non-zero magnetic moment in solution, such impurities are not present in the ESI-MS spectra, which gave *m*/*z* values of 1077.6760 (ESI(+)) and 1077.6251 (ESI(−)), with the highest intensity. This would thus indicate the detection of [M + H]^+^ and [M + H]^−^ ions, while the calculated exact mass for the complex based on the X-ray studies is 1076.6323. The MS patterns were also rather atypical for a vanadium complex (see Supplementary Information), which may very well be the manifestation of the redox behavior of the complex. In addition, the positive ionization mode gives traces of [M + Na]^+^ and [M + K]^+^ ions that are detected at *m*/*z* = 1099.6508 and 1115.6145, respectively.

[Ni(HL^ox^)_2_] proved to be paramagnetic in the NMR measurements, so the Evans method was applied to measure µ_eff_ = 2.84 µ_B_ at room temperature. The magnetic properties were further studied in the solid state using SQUID experiments (see below). The protonation state of the coordinated ligands was also verified using ESI-MS, while the molecular ion [M + H]^+^ was detected at *m*/*z* = 1085.6731.

### 2.4. DFT Studies for the Vanadium Complex

Density functional theory (DFT) was employed to investigate the electronic structure of the [V(L)(HL)] complex. DFT calculations were carried out using PBE0 functional [27,28,29] and def2-TZVP basis sets [30] (with def2/J auxiliary basis sets [31]) on a modified structure of [V(L)(HL)] with *tert*-butyl groups replaced with H atoms. Geometry optimizations were carried out within the Orca program (version 4.2.0) [32] for singlet, broken symmetry singlet, triplet and pentet electronic states, using the modified single crystal X-ray structure as a starting point. The optimized structures were then subjected to vibrational analyses to establish that they each correspond to a minimum on the potential energy surface.

From the studied systems, a broken symmetry (BS) singlet (*S* = 0) was found to correspond to the ground state, with the triplet (*S* = 1) only 6 kJ/mol higher in energy, followed by the closed shell singlet (*S* = 0; 22 kJ/mol higher than BS singlet). The optimized structure of the pentet (*S* = 2) was found to be considerably less favorable (51 kJ/mol higher in energy). The coordination sphere bond lengths (Table S2) of the ground state broken symmetry solution fall within ±0.03 Å from the corresponding experimental values (complex A in the asymmetric unit). The triplet structure shows a slightly larger deviation, whereas, interestingly, the closed shell singlet solution shows the best fit for experimental metal-ligand bond lengths. Furthermore, the asymmetry of the V-N bonds is best reproduced by the closed shell singlet optimized geometry. The xyz coordinates of the optimized structures are given in Supplementary Materials, Table S4.

**Figure 3:**
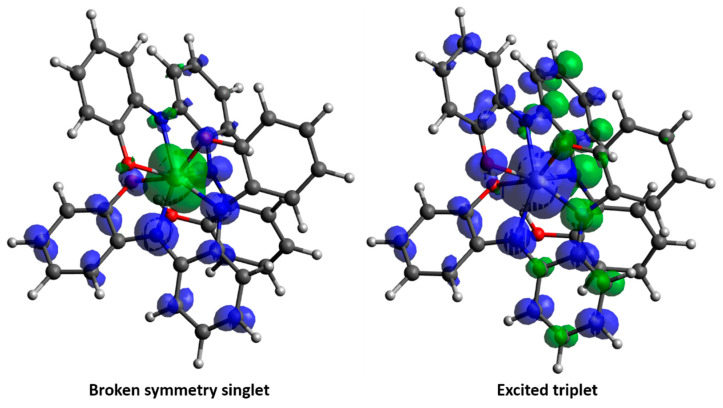
Spin density plots for broken symmetry singlet and excited triplet states of modified structure of [V(L)(HL)].

Analysis of the spin density of the broken symmetry singlet shows the localization of the opposite spins to the vanadium center and, respectively, to the tetradentate ligand with only a minute contribution from the tridentate ligand (Figure 3). The visual information together with the analysis of the Löwdin atomic spin populations strongly point to an antiferromagnetically coupled radical ligand and vanadium(4+) center as the ground state structure. The low-lying excited triplet state shows the spin localized, to a great extent, to the vanadium ion, but also delocalized along the ligand backbones. According to the Löwdin atomic spin populations the triplet contains a vanadium(III) ion, and thus, the ligands should exist at either [L^ox^]^2−^ and [HL^ox^]^−^ or [L^sq1^]^3−^ and [HL^sq2^] (or [HL^sq1^]^2−^ and [L^sq2^]^−^) oxidation states, respectively. From these formal oxidation states, the former has no unpaired electrons, whereas the latter oxidation states would require the assignment of half a spin to each of the ligands. As there is significant spin density on both ligands, the results indicate that the ligands of the excited triplet state structure can be assigned as [L^sq1^]^3−^ and [HL^sq2^] (or [HL^sq1^]^2−^ and [L^sq2^]^−^).

### 2.5. Electrochemical Studies

**Figure 4:**
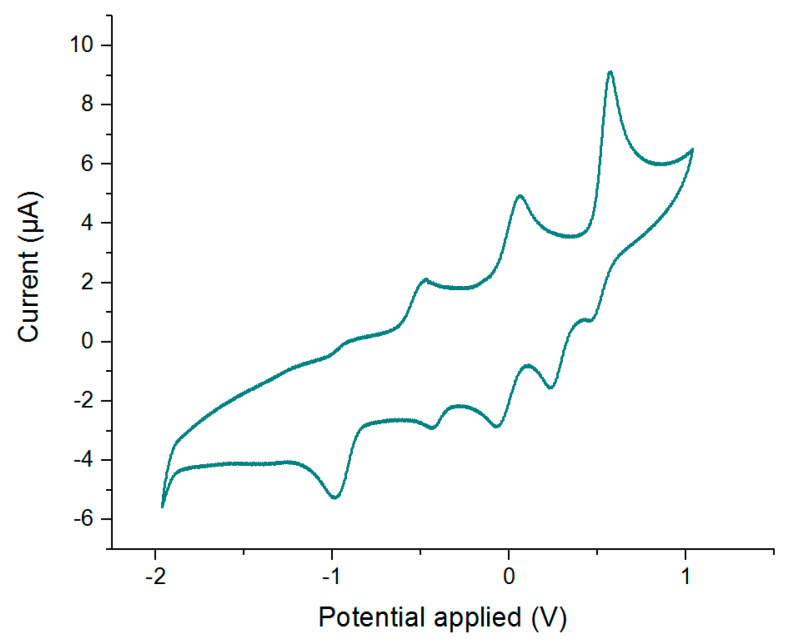
Cyclic voltammogram of [V(L^sq1^)(HL^ox^)]. Cyclic voltammetry is performed at RT vs Fc/Fc^+^, in CH_2_Cl_2_ with 100 mVs^−1^ scan rate and the rotation of the measurement is clockwise.

Cyclic voltammetry was used to study the redox stability and the characteristic redox behavior of [Ti(L^ox^)_2_] and [Zr(L^ox^)_2_] by scanning the potential range from +1.3 to −2.3 V vs Fc^+^/Fc. A different range, from +1.0 to −2.0 V vs Fc^+^/Fc, was applied for [V(L^sq1^)(HL^ox^)] and [Ni(HL^ox^)_2_]. The reversible electron transfer processes for Ti and Zr and Ni complexes are probably only ligand based [5,6,8], as similar waves can be observed in the voltammograms of each of the compounds. Some of the redox processes are at overlapping potentials, or faint, thus rendering the voltammograms somewhat difficult to interpret. The voltammogram of the V complex, on the other hand, is clearly different. (Figure 4, Table 3, Supplementary Materials Figures S7–S10) The voltammogram exhibits two reversible one-electron processes (+0.01 and +0.54 V) and several irreversible processes in the mix. It is therefore difficult to draw definite conclusions regarding which redox event takes place at each of the redox waves of the voltammogram.

### 2.6. Optical Absorbtion Studies

**Figure 5:**
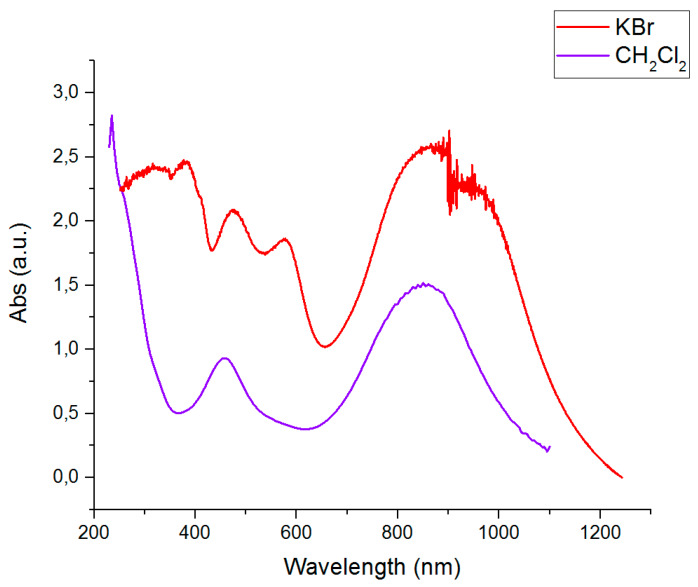
The UV-vis-NIR spectra of [Ni(HL^ox^)_2_].

One of our motivations for the synthesis of the presented compounds was their potential applicability in photovoltaics as dyes, and thus, their optical absorption properties were studied initially using UV-vis spectroscopy. Accordingly, the UV-vis-NIR spectra in CH_2_Cl_2_ present distinct absorption peaks for each complex in the vis/NIR range (Table 4, Figure 5). The absorption coefficients of the different complexes range from low to significantly high when compared with the coefficient of the standard ruthenium sensitizer dye (ε ≈ 14 × 10^3^ M^−1^cm^−1^, λ = 538 nm) [33]. The absorption spectra are rather similar in shape in the solid state, as well in CH_2_Cl_2_ solution for all complexes (see Supplementary Materials). The absorption coefficients of the different complexes range from low to significantly high when compared with the coefficient of the standard ruthenium sensitizer dye (ε ≈ 14 × 10^3^ M^−1^cm^−1^, λ = 538 nm) [33]. The absorption spectra are rather similar in shape in the solid state, as well in CH_2_Cl_2_ solution for all complexes (see Supplementary Materials). The group IV complexes have similar, ligand-based, π→π* UV absorptions as the ones that have been previously reported [4]. The strong NIR absorptions above 1000 nm are most probably ligand-based transitions, i.e., ligand-to-ligand charge transfer (LLCT) in character, as reported for the related Co complex [7]. The LLCT processes are of both the intra and inter ligand type [34]. As these metal centers are high oxidation state species with empty d-orbitals, there may be some ligand-to-metal charge transfer (LMCT) involved. [V(L^sq1^)(HL^ox^)] has a broad absorption at λ_max_ = 680 nm, which reaches the NIR range. This absorption may originate from LLCT with some LMCT or metal-to-ligand CT (MLCT) mixing. As the central metal is not d^0^, also a d-d transition is possible. In addition, the broad and intense absorption at 850 nm for [Ni(HL^ox^)_2_] is assignable as a LLCT band and/or to the Ni d-d absorptions.

### 2.7. EPR Studies for the Vanadium and Nickel Complexes

**Figure 6:**
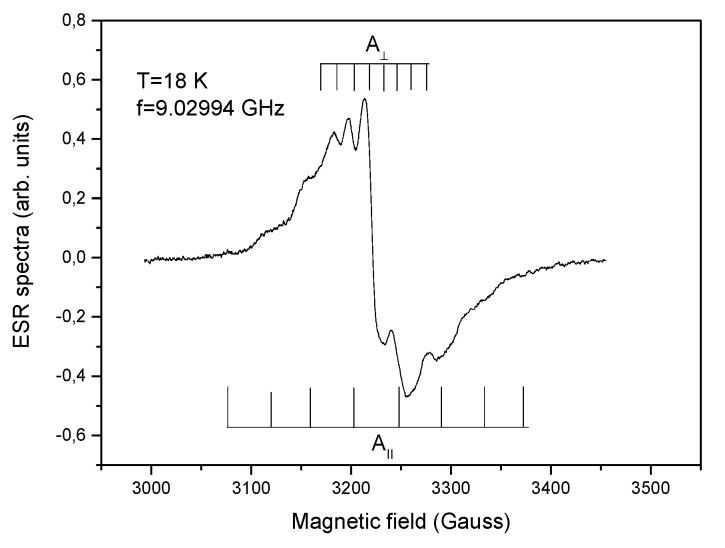
The EPR spectrum of powder [V(L^sq1^)(HL^ox^)] sample.

EPR (Electron Paramagnetic Resonance) studies were conducted to examine the electronic nature of the V and Ni complexes more closely. The Ni complex, having an integer spin S = 2/2, measured in its powder form, was found to be EPR silent. The spectrum of [V(L^sq1^)(HL^ox^)] is described by Hamiltonian for electron spin S:

H = β B g S + S A I,(1)

where β is Bohr magneton, B is a magnetic field, g and A are the tensors of axial symmetry of g-factor and hyperfine interaction between electron and ^51^V (spin I = 7/2) nuclei, respectively. Two sets of partly resolved lines in spectrum shown in Figure 6 correspond to hyperfine interaction between electron and ^51^V nuclei for different values of the parallel and perpendicular components of A-tensor: A_||_=42 G (or 117 MHz, or 39 10^−4^ cm^−1^) and A_⊥_=16 G (or 45 MHz, or 15 10^−4^ cm^−1^). The values of g-tensor components are g_||_=2.001 and g_⊥_=1.999. The estimated hyperfine structure splitting is about two to three times lower compared to previously reported V^4+^ ESR spectra [35,36,37,38,39]. This shows that the localization of the spin density on the ^51^V nuclei is lower in the investigated samples. The g-factor components are also higher with respect to reported earlier data, which further supports the idea of a delocalized metal-organic radical system.

### 2.8. Magnetic Properties

The magnetic nature and thermal dependency of the complexes’ magnetic properties were investigated using a SQUID magnetometer in the temperature range of 2–300 K. The Ti and Zr complexes exhibit diamagnetic properties in the SQUID measurements, as expected. The possibility of utilizing synthesized complexes in photovoltaic applications was ascertained by observing the magnetic properties with or without irradiation in the NIR region (a laser diode operating at λ = 785 nm), and it was confirmed that the Ti and Zr complexes are not able to transform the absorbed NIR photons to free electrons. The measurements suggest that the magnetic moment, and therefore the number of unpaired spins, remains unchanged within the molecule when the complexes [TiL^ox^)_2_] and [ZrL^ox^)_2_] are illuminated.

**Figure 7:**
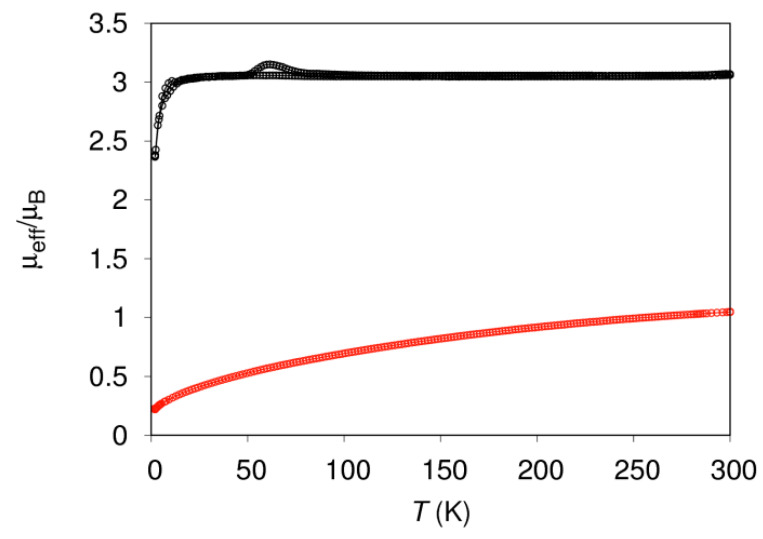
The measured effective magnetic moments for [V(L^sq1^)(HL^ox^)] (bottom) and [Ni(HL^ox^)_2_] (top) in the function of temperature. The signal observed in the right figure at ca. 70 K is an instrumental artefact.

**Figure 8:**
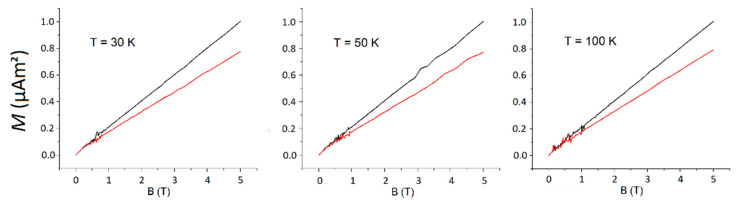
The measured virgin curves of [V(L^sq1^)(HL^ox^)] on a SrTiO_3_ disk. The red curves result from the measurements done under irradiation in the NIR region, and the black curves are from the measurements done without irradiation.

[V(L^sq1^)(HL^ox^)] gave a paramagnetic signal in SQUID measurements. The temperature dependence of the magnetic susceptibility was also measured, and the data were fitted to the Curie law of localized moments. The 1/χ_mol_ curve does not follow the Curie law, which indicates that there is some electron spin density delocalized on the ligand rather than on the metal center. The measured effective magnetic moment is low (Figure 7) and similar in magnitude to the µ_eff_ measured in solution using the Evans method. Although we cannot unambiguously show that no magnetic impurities are present, we propose that the measured magnetic moments, both in a solution and in a solid state, suggest that [V(L^sq1^)(HL^ox^)] consists prevalently of an antiferromagnetically coupled radical ligand and V^4+^ metal ion with one unpaired electron. The paramagnetic signal, which is shown in the molar magnetic susceptibility plot, is then due to a low-lying triplet state which is thermally populated, as suggested by the DFT calculcations. The field dependence curve of [V(L^sq1^)(HL^ox^)] is typical for a paramagnetic complex and no hysteresis is observed (see Supplementary Materials). By observing the magnetic properties with or without irradiation in the NIR region (a laser diode operating at λ = 785 nm), it was possible to confirm that the complex is able to transform the absorbed NIR photons to paired electrons. The measurements (Figure 8) suggest that the magnetic moment, and therefore the number of unpaired spins, is decreased within the molecule when the complex [V(L^sq1^)(HL^ox^)] is illuminated. The effect was not permanent at any temperature, suggesting that the complex could be used to continuously convert IR quanta to paired electrons.

**Figure 9:**
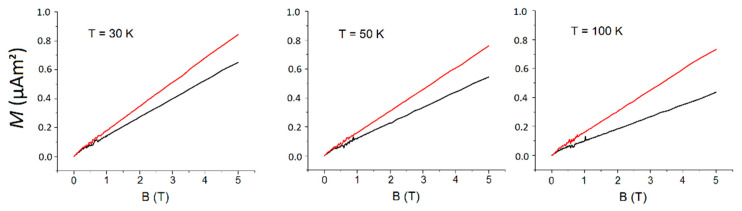
The measured virgin curves of [Ni(HL^ox^)_2_] on a SrTiO_3_ disk. The red curves correlate to the measurements done under irradiation in the NIR region, and the black curves resemble the measurements done without irradiation.

The [Ni(HL^ox^)_2_] complex, as expected, also gave a paramagnetic signal. The temperature dependence of the susceptibilities was also measured, and the data were fitted to the Curie law of localized moments. The 1/χ_mol_ plot followed the Curie law, and the effective magnetic moment for the molecule was calculated from the slope of the plot. The result was µ_eff_ = 2.88 µ_B_, which is in good agreement with an octahedral high-spin Ni^2+^ moiety. The field dependence curve of [Ni(HL^ox^)_2_] is typical for a paramagnetic complex. The Ni complex was found to react under NIR radiation, but slightly differently than the V complex. The Ni complex converted photons to free electrons, thus increasing its magnetic moment (Figure 9). As in the studies with the V complex, the effect was not permanent at any studied temperature.

In conclusion, a series of transition metal complexes was made using a redox non-innocent *N*,*N*’-bis(3,5-di-tertbutyl-2-hydroxy-phenyl)-1,2-phenylenediamine H_4_L as a ligand precursor. H_4_L can form complexes with a 2:1 ligand-to-metal ratio with different transition metal precursors. In these complexes, two organic molecules coordinate to the metal centre as tri- or tetradentate ligands and display a range of different oxidation states.

Several group IV metal complexes with L ligand were previously prepared by the reaction of metal tetrachlorides with Li_4_L to yield molecular complexes [M(L^red^)(S)_n_] (M = Ti, Zr, Hf; S = pyridine, THF; *n* = 2,3), which can be further oxidised by PhICl_2_ to yield corresponding chlorides [MCl_2_(L^ox^)(S)_n_] [3,4]. Furthermore, the reaction of H_4_L with [M(O-*i*-Pr)_4_] (M = Ti, Zr) leads to the formation of either [Ti(L^Ox^)(O-*i*-Pr)_2_] or [{µ-L^red^}(M(µ-O-*i*-Pr)(O-*i*-Pr)_2_)_2_], depending on the stoichiometry [2]. In the present study we show that under ambient conditions, where the organic proligand is abundantly available, rather than being added dropwise to metal precursor solution, 2:1 ligand-to-metal complex is formed.

The essence of the electronic structure of the V complex remained somewhat unclear after many different analyses. However, preliminary DFT calculations, carried out on a model complex with *tert*-butyl substituents replaced by H atoms, predict a broken symmetry singlet ground state for [V(L)(HL)]. Analysis of spin density shows a localization of opposite spins on the V center and the tetradentate ligand, respectively, with negligible contribution from the tridentate ligand. This corresponds to the antiferromagnetically coupled [V(L^sq1^)(HL^ox^)] structure with V^4+^, suggested also on the basis of metrical oxidation states. However, the broken symmetry singlet ground state and the lowest triplet state are separated only by ca. 6 kJ/mol, with a closed shell singlet a further 16 kJ/mol higher in energy. The existence of several electronic states close in energy suggests that multiconfigurational methods would be required to depict the electronic structure of [V(L^sq1^)(HL^ox^)] accurately. From the DFT results, it can be deduced, however, that the experimentally observed magnetism is likely due to mixing of several low-lying singlet and triplet electronic states.

All compounds used in this study have strong NIR absorptions and some show very interesting magnetic and electrochemical properties. While strong NIR absorbers, the Ti and Zr complexes regretfully did not produce any magnetic behavior when illuminated. This was probably due to the diamagnetic nature of the complexes. Nevertheless, the Ti and Zr complexes still pose an interesting comparison with the V and Ni complexes that generate (un)paired electrons under NIR radiation. This indicates great promise in the photovoltaic applications and furthers our knowledge regarding NIR-absorbing complexes, their magnetic behavior and their possible photovoltaic applications. Further investigation of these compounds is currently ongoing in our group.

## 3. Materials and Methods

The starting complex [Ni(acac)_2_·2H_2_O] was synthesized by the well-known reaction of Ni(NO_3_)_2_·6H_2_O with the excess of 2,4-pentanedione in an aqueous solution using sodium acetate as a base. The product was filtered and washed with water. The ligand precursor was synthesized using literature procedures [4,8]. Other chemicals were used as purchased from commercial sources. The solvents used were of HPLC grade. All syntheses were done under an ambient atmosphere.

The ^1^H and ^13^C-NMR spectra were recorded with 500 Mhz Bruker AVANCE-III NMR system. ESI-MS spectra for complexes were measured in the positive-ion mode with a Bruker micrOTOF-Q spectrometer. The samples were injected as dichloromethane-acetonitrile solutions. Cyclic voltammetry (CV) for complexes were recorded at ambient temperature using a platinum working electrode, a 1 mm diameter platinum counter electrode, and a Ag/AgCl reference electrode. Samples were dissolved in CH_2_Cl_2_ containing 0.1 M (Bu_4_N)ClO_4_ as the supporting electrolyte. The voltammograms were recorded at a scan rate of 100 mV/s, while the potentials were measured in volts versus the Fc^+^/Fc couple. The thermal changes of the complex were studied with TA Instrument SDT Q600 simultaneous TGA-DSC apparatus between 23 and 500 °C in flowing nitrogen gas using an aluminum oxide pan as sample holder and reference. A flow rate of 100 mL/min and a heating rate of 5 °C/min were applied. Amounts of [Ti(L^ox^)_2_], [Zr(L^ox^)_2_], [V(L^sq1^)(HL^ox^)] and [Ni(HL^ox^)_2_] were 7.94, 10.89, 4.77 and 4.48 mg, respectively. UV-Vis spectra in CH_2_Cl_2_ solution were measured with an Agilent Cary 60 UV/vis spectrophotometer in a 10 mm quartz glass cuvette. UV-Vis-NIR spectra were measured of KBr pellets with a Varian Cary 50 spectrophotometer, and a blank KBr pellet was used as reference. The solid-state powder of the [V(L^sq1^)(HL^ox^)] sample ESR spectrum was recorded at 18 K using a ESR spectrometer operating at the microwave frequency of 9.030 GHz and 100 kHz modulation of magnetic field. A look-in amplifier was used for observing the first derivative shape of ESR absorption lines under magnetic field scan. The temperature of samples was controlled using a He-gas flow cryostat. The magnetic properties were measured in a SQUID magnetometer. The temperature dependence of the zero-field-cooled (ZFC) and field-cooled (FC) magnetization was measured between temperatures of 2 and 300 K with a Quantum Design SQUID magnetometer MPMS XL with external magnetic field of B = 1.0 T on 7.01, 7.67, 35.6 and 10.7 mg samples of [Ti(L^ox^)_2_], [Zr(L^ox^)_2_], [V(L^sq1^)(HL^ox^)] and [Ni(HL^ox^)_2_], respectively, sealed in plastic nonmagnetic straws. The field dependence was measured at 2 K between −2.5 and 2.5 T using 50 mT steps. Virgin magnetizations as a function of B and magnetic hysteresis curves were recorded in magnetic fields up to 5 T at temperatures of 2, 30, 50 and 100 K. Although the magnetic particles were randomly oriented on the surface of the substrate, the external magnetic field B was always oriented along the out-of-plane axis of the substrate. The photoinduced magnetization measurements were performed in dark or under illumination through an optical fiber with a home-made fiberoptic sample holder attached to the SQUID magnetometer. The samples of [Ti(L^ox^)_2_], [Zr(L^ox^)_2_], [V(L^sq1^)(HL^ox^)] and [Ni(HL^ox^)_2_] were drop casted as diethyl ether solutions on circular SrTiO_3_ plates (diameter 5 mm). The light source was a Fabry-Perot laser diode by Thorlabs operating at λ = 785 nm (1.58 eV) with a maximum output power of 10 mW measured at the end of the optical fiber, and hence, the laser fluence on the sample surface was ca. 0.5 mW/mm^2^. The single crystal X-ray diffraction (XRD) analyses were carried out using an Agilent SuperNova microfocus dual source (Cu/Mo) diffractometer equipped with an Atlas detector. The data collection and reduction, including multifaceted crystal model-based analytical absorption correction, were carried out using the CrysAlis^pro^ program [40]. The structures were solved with ShelXS [41] using direct methods and refined on *F*^2^ using full matrix least squares techniques with ShelXL [42] within the Olex^2^ (v. 1.2) [43] program package. All non-hydrogen atoms were refined anisotropically. The hydrogen atoms were refined using a riding model with fixed thermal parameters 1.2–1.5 times the values of the corresponding host atoms (the O–H distances of the O2 and O4 hydroxyl groups were refined freely).

### 3.1. Preparation of [Ti(L^ox^)_2_]

A total of 44.2 mg (0.276 mmol) of TiOSO_4_·H_2_O with the ligand H_4_L (156 mg, 0.300 mmol) were dissolved in methanol (30 mL), and a base, Et_3_N (0.170 mL), was added. The reaction mixture turned slowly to turn dark green at room temperature and it was left to stand for 2 days. The reaction mixture was evaporated to dryness, washed with hexane and then filtered. The filtrate was evaporated and filtered through a silica plug using toluene as an eluent. The toluene filtrate was evaporated to dryness and the amount of dry product was 43.1 mg (yield 27%). [Ti(L^ox^)_2_] dissolves moderately in alcohols and acetonitrile, whereas it is readily soluble in diethyl ether, halogenated organic solvents and hydrocarbon solvents. X-ray diffraction quality crystals of [Ti(L^ox^)_2_]·CH_3_CN were obtained from acetonitrile solution with slow evaporation. IR: 2952 (s), 2922 (s), 2856 (s), 1597 (w), 1541 (w), 1462 (s), 1439 (s), 1379 (m), 1359 (m), 1322 (s), 1290 (w), 1256 (vs), 1193 (w), 1167 (vs), 1152 (s), 1131 (s), 1113 (vs), 1023 (m), 995 (m), 914 (m), 890 (m), 867 (m), 846 (m), 802 (m), 778 (w), 740 (vs), 667 (m), 644 (w), 633 (m), 598 (m), 585 (m), 569 (m), 539 (w), 508 (s), 481 (w) cm^−1^. ^1^H-NMR (CDCl_3_): δ 7.55 (dd, *J*_HH_ = 3.30 and 7.39 Hz, 4H, Ph-H), 7.23 (d, *J*_HH_ = 1.82 Hz, 4H, Ph-H), 6.98 (s, 4H, Ph-H), 6.95 (dd, *J*_HH_ = 3.11 and 7.43 Hz, 4H, Ph-H), 1.22, 0.92 (2s, 72H, C(CH_3_)_3_), ppm. ^13^C-NMR (CDCl_3_): δ 166.91, 150.95, 140.23, 137.10, 130.21, 126.10, 124.25 (Ph-C) 34.41, 34.24 (C*(CH_3_)_3_), 31.37, 29.15 (C(C*H_3_)_3_) ppm. ESI(+)-MS: *m/z* 1073.62 ([M + H]^+^ calcd. *m/z* 1073.64). m.p. 270 °C (decomp., measured for [Ti(L^ox^)_2_]·CH_3_CN).

### 3.2. Preparation of [Zr(L^ox^)_2_]

Methanol solution (20 mL) of ZrOCl_2_·8 H_2_O (104 mg, 0.323 mmol) was introduced to the solid ligand H_4_L (144 mg, 0.279 mmol) and Et_3_N (0.160 mL). The reaction mixture gradually changed its color from neutral to very intense blue. The closed reaction vessel was left to stand overnight at room temperature to obtain large, X-ray diffraction quality single crystals of [Zr(L^ox^)_2_]. The crystals were isolated by filtration and washed with methanol to obtain 69.6 mg (44%) of solid product. [Zr(L)_2_] is insoluble in alcohols and acetonitrile, but soluble in diethyl ether, halogenated organic solvents and hydrocarbon solvents. IR: 2953 (s), 2905 (s), 2868 (s), 1531 (s), 1461 (s), 1439 (s), 1398 (m), 1385 (m), 1360 (s), 1320 (m), 1289 (m), 1251 (vs), 1192 (s), 1163 (vs), 1148 (s), 1129 (vs), 1113 (vs), 1023 (m), 992 (w), 982 (w), 913 (w), 893 (m), 868 (w), 849 (m), 816 (m), 780 (w), 739 (s), 661 (m), 644 (m), 632 (m), 597 (m), 582 (m), 565 (m), 538 (w), 504 (s) cm^−1^. ^1^H-NMR (CDCl_3_): δ 7.57 (dd, *J*_HH_ = 3.18 and 7.45 Hz, 4H, Ph-H), 7.28 (d, *J*_HH_ = 1.89 Hz, 4H, Ph-H), 7.00 (s, 4H, Ph-H), 6.97 (dd *J*_HH_ = 3.13 and 7.33 Hz, 4H, Ph-H), 1.23, 0.91 ppm (2s, 72H, C(CH_3_)_3_) ^13^C NMR (CDCl_3_): δ 167.11, 152.02, 140.62, 130.51, 124.48, 115.66 (Ph-C), 34.49, 34.39 (C*(CH_3_)_3_), 31.30, 29.04 (C(C*H_3_)_3_), ppm. ESI(+)-MS: *m/z* 1115.62 ([M + H]^+^ calcd. *m/z* 1115.59). ESI(+)-MS: *m/z* 1137.60 ([M + Na]^+^ calcd. *m/z* 1137.58). ESI(+)-MS: *m/z* 1153.57 ([M + K]^+^ calcd. *m/z* 1153.55). m.p. 340 °C (decomp.).

### 3.3. Preparation of [V(L^sq1^)(HL^ox^)]

A total of 152 mg (0.294 mmol) of H_4_L was added into a methanol solution (8 mL) which contained 79.4 mg (0.314 mmol) of [VOSO_4_·5H_2_O] and 50 µL of NEt_3_. The reaction mixture turned dark purple quite swiftly and the reaction was left to run in a closed vessel for 3 days at room temperature. The reaction mixture was evaporated to dryness and the product was separated by silica column chromatography using diethyl ether as eluent. The yield was 106 mg (67%). The isolated solid dissolves in alcohols and acetonitrile to some extent, while halogenated organic solvents, diethyl ether and hydrocarbon solvents dissolve the complex with ease. X-ray diffraction quality single crystals were obtained directly from the MeOH reaction mixture. IR: 2951 (s), 2904 (s), 2867 (s), 1582 (w), 1446 (s), 1415 (m), 1391 (m), 1360 (s), 1334 (m), 1249 (vs), 1196 (s), 1160 (s), 1136 (s), 1112 (s), 1025 (m), 989 (m), 911 (m), 862 (w), 821 (w), 770 (w), 740 (s), 665 (m), 647 (m), 602 (w), 586 (w), 545 (m), 506 (m), 463 (m), cm^−1^. ^1^H-NMR (CDCl_3_): δ 1.53, 1.50, 1.29, 1.01 ppm (4s, 72H, C(CH_3_)_3_). ESI(+)-MS: *m/z* 1077.65 ([M]^+^ calcd. *m/z* 1076.63). ESI(+)-MS: *m/z* 1099.65 ([M + Na]^+^ calcd. *m/z* 1099.62). ESI(+)-MS: *m/z* 1115.61 ([M + K]^+^ calcd. *m/z* 1115.60). ESI(−)-MS: *m/z* 1077.64 ([M]^−^ calcd. *m/z* 1076.63). m.p. 250 °C (decomp.). Evans method, µ_eff_ = 0.66 µ_B_.

### 3.4. Preparation of [Ni(HL^ox^)_2_]

[Ni(acac)_2_·2H_2_O] (123 mg, 0.420 mmol) and H_4_L (446 mg, 0.863 mmol) were stirred in methanol (10 mL) for 21 h. Precipitated dark solid was filtered and washed with methanol, fractioned with column chromatography using CH_2_Cl_2_ as an eluent and finally recrystallized from the Et_2_O/MeOH mixture to obtain XRD quality crystals. The isolated solid is insoluble in alcohols or acetonitrile, but dissolves in halogenated organic solvents, diethyl ether and hydrocarbon solvents. Yield 109 mg (23 %). IR: 2954 (m), 1442 (m), 1358 (s), 1253 (vs), 1193 (m), 1166 (s), 1131 (s), 1113 (s) 1022 (m), 985 (m), 908 (m), 868 (w), 833 (w), 756 (m), 598 (m) 580 (m), 552 (m), 501 (s) cm^−1^. ^1^H-NMR (CDCl_3_): δ 0.7–2.1 ppm (72 H, several overlapping peaks). ESI(+)-MS: *m/z* 1085.6731 ([M + H]^+^ calcd. *m/z* 1085.6349). m.p. 230 °C (decomp.). Evans method, µ_eff_ = 2.84 µ_B_.

## Figures and Tables

**Table 1:** Selected bond lengths (Å) for [Ti(L^ox^)2] and [Zr(L^ox^)2].

Ti1A-O1A	1.977(2)	Zr1A-O1A	2.113(4)
Ti1A-O2A	2.019(2)	Zr1A-O2A	2.131(4)
Ti1A-O3A	2.016(2)	Zr1A-O3A	2.096(3)
Ti1A-O4A	2.022(2)	Zr1A-O4A	2.134(3)
Ti1A-N7A	2.318(2)	Zr1A-N7A	2.382(4)
Ti1A-N14A	2.198(3)	Zr1A-N14A	2.325(4)
Ti1A-N27A	2.282(3)	Zr1A-N27A	2.367(4)
Ti1A-N34A	2.228(3)	Zr1A-N34A	2.319(4)
N7A-C6A	1.379(4)	N7A-C6A	1.377(7)
N7A-C8A	1.333(4)	N7A-C8A	1.333(6)
N14A-C13A	1.327(4)	N14A-C13A	1.346(6)
N14A-C15A	1.377(4)	N14A-C15A	1.379(6)
N27A-C26A	1.361(4)	N27A-C26C	1.399(13)
N27A-C28A	1.350(4)	N27A-C28A	1.342(6)
N34A-C33A	1.340(4)	N34A-C33A	1.340(6)
N34A-C35A	1.365(4)	N34A-C35A	1.387(6)

**Table 2:** Selected bond lengths (Å) for [V(L^sq1^)(HL^ox^)] and [Ni(HL^ox^)2].

V1A-O1A	3.571(2)	Ni1-O1	2.0616(16)
V1A-O2A	1.9712(19)	Ni1-O2	3.548(2)
V1A-O3A	1.9496(19)	Ni1-O3	2.0315(14)
V1A-O4A	2.0544(19)	Ni1-O4	3.476(2)
V1A-N7A	2.071(2)	Ni1-N7	1.9924(16)
V1A-N14A	2.155(2)	Ni1-N14	2.1021(17)
V1A-N27A	2.052(2)	Ni1-N27	1.9882(16)
V1A-N34A	1.989(2)	Ni1-N34	2.0824(16)
N7A-C6A	1.425(3)	N7-C6	1.375(3)
N7A-C8A	1.322(4)	N7-C8	1.335(3)
N14A-C13A	1.323(4)	N14-C13	1.323(2)
N14A-C15A	1.362(4)	N14-C15	1.414(3)
N27A-C26A	1.397(4)	N27-C26	1.363(3)
N27A-C28A	1.392(4)	N27-C28	1.308(3)
N34A-C33A	1.371(4)	N34-C33	1.310(2)
N34A-C35A	1.402(3)	N34-C35	1.415(2)

**Table 3:** Measured redox potentials for studied complexes (vs Fc^+^/Fc).

Complex	Reversible One-Electron Redox Waves (V)					
[Ti(L^ox^)_2_]	−1.64	−1.19	−0.17	+0.24	+1.06	
[Zr(L^ox^)_2_]	−1.95	−1.49	−1.09	−0.02	+0.41	+0.74
[V(L^sq1^)(HL^ox^)]			+0.01	+0.54		
[Ni(HL^ox^)_2_]	−1.47	−1.07	−0.92	+0.04	+0.36	

**Table 4:** The UV-vis-NIR absorption peaks in CH_2_Cl_2_ solution and their molar absorption coefficients.

Complex	λ_max_ (nm)	ε (10^3^ × L mol^−1^ cm^−1^)
[Ti(L^ox^)_2_]	390	4.3
	570 (sh)	2.6
	710	5.3
	1050	2.2
[Zr(L^ox^)_2_]	285	19
	370	14
	760	13
	1020	8.5
[V(L^sq1^)(HL^ox^)]	315	16
	430	7.8
	540 (sh)	8.1
	680	13
[Ni(HL^ox^)_2_]	460	14
	850	23

## Supplementary Materials

The supplementary material is available online at https://www.mdpi.com/1420-3049/25/11/2531/s1. Table S1, S2: Summary of crystallographic data. Figure S1: Molecular structure of orthorhombic polymorph of [V(Lsq1)(HLox)]. Figure S2: The experimental and simulated PXRD pattern of [V(Lsq1)(HLox)]. Figures S3–S6: UV-Vis-NIR spectra. Figures S7–S10: Cyclic voltammograms. Figures S11-S16: ESI-MS mass spectra. Figures S17–S18: SQUID measurements. Figures S19–S22: TGA/DSC measurements. Figures S23–S28: NMR spectra. Figures S29–S30: Evans’ method NMR measurements. Table S2: Experimental and optimized (DFT) bond parameters. Table S3 Optimized Cartesian coordinates.
