# Supplementary material for: Series of Near-IR-Absorbing Transition Metal Complexes with Redox Active Ligands
Source: Molecules. 2020 May 29;25(11):2531. doi: 10.3390/molecules25112531 (PMC7321360; doi:10.3390/molecules25112531)
Supplement: Supplementary file 1 [file molecules-25-02531-s001.pdf]

Supplementary Materials

Series of Near-IR-Absorbing Transition Metal Complexes with Redox Active Ligands

Esko Salojärvi [1], Anssi Peuronen [1], Manu Lahtinen [2], Hannu Huhtinen [3], Leonid S. Vlasenko [3,4], Mika Lastusaari [1] and Ari Lehtonen [1,*]

[1] Inorganic Materials Chemistry research group, Department of Chemistry, University of Turku, FI-20014 Turku, Finland; emsalo@utu.fi (E.S.); anssi.peuronen@utu.fi (A.P.); miklas@utu.fi (M.L.)
[2] Department of Chemistry, P.O. Box 35, University of Jyvaskyla, FI-40014 Jyvaskyla, Finland; manu.k.lahtinen@jyu.fi
[3] Wihuri Physical Laboratory, Department of Physics and Astronomy, University of Turku, FI-20014 Turku, Finland; hannu.huhtinen@utu.fi (H.H.); leonidvlasenko@yahoo.com (L.S.V.)
[4] Ioffe Institute, Russian Academy of Sciences, St. Petersburg 194021, Russia
* Correspondence: arileh@utu.fi

Content:

Table S1: Summary of crystallographic data for [Ti(L^{ox})$_2$], [Zr(L^{ox})$_2$], [Ni(HL^{ox})$_2$] and [V(L^{sq1})(HL^{ox})].

Table S2: Summary of crystallographic data for polymorphic crystal structure of [V(L^{sq1})(HL^{ox})].

Figure S1: Illustration of molecular structure of orthorhombic polymorph of [V(L^{sq1})(HL^{ox})] measured by single crystal XRD.

Figure S2: Comparison between the experimental PXRD pattern of [V(L^{sq1})(HL^{ox})] and monoclinic and orthorhombic polymorphs.

Figures S3-S6: UV-vis-NIR spectra for [Ti(L^{ox})$_2$], [Zr(L^{ox})$_2$], [V(L^{sq1})(HL^{ox})] and [Ni(HL^{ox})$_2$].

Figures S7-S10: Cyclic voltammograms for [Ti(L^{ox})$_2$], [Zr(L^{ox})$_2$], [V(L^{sq1})(HL^{ox})] and [Ni(HL^{ox})$_2$].

Figures S11-S16: ESI-MS mass spectra for [Ti(L^{ox})$_2$], [Zr(L^{ox})$_2$], [V(L^{sq1})(HL^{ox})] and [Ni(HL^{ox})$_2$].

Figures S17-S18: SQUID measurements for [V(L^{sq1})(HL^{ox})] and [Ni(HL^{ox})$_2$].

Figures S19-S22: TGA/DSC measurements for [Ti(L^{ox})$_2$], [Zr(L^{ox})$_2$], [V(L^{sq1})(HL^{ox})] and [Ni(HL^{ox})$_2$].

Figures S23-S28: ^{1}H and ^{13}C NMR measurements for [Ti(L^{ox})$_2$], [Zr(L^{ox})$_2$], [V(L^{sq1})(HL^{ox})] and [Ni(HL^{ox})$_2$].

Figures S29-S30: Evans' method NMR measurements for [V(L^{sq1})(HL^{ox})] and [Ni(HL^{ox})$_2$].

Table S2 Comparison between selected experimental and optimized (DFT) bond parameters

Table S3 Optimized cartesian coordinates for [(V(L)(HL)] from DFT calculations (in Å)

Table S1. Summary of crystallographic data for [Ti(L^{ox})$_2$], [Zr(L^{ox})$_2$], [Ni(HL^{ox})$_2$] and [V(L^{sq1})(HL^{ox})].

**Complex**	**[Ti(L^{ox})$_2$]·CH_3CN**	**[Zr(L^{ox})$_2$]**	**[V(L^{sq1})(HL^{ox})]**	**[Ni(HL^{ox})$_2$]**
Empirical formula	$C_{70}H_{91}N_5O_4Ti$	$C_{68}H_{88}N_4O_4Zr$	$C_{68}H_{89}N_4O_4V$	$C_{68}H_{90}N_4NiO_4$
Formula weight	1114.37	1116.64	1077.37	1086.14
Temperature/K	120.0(1)	120.0(1)	175.0(1)	120.0(1)
Crystal system	triclinic	triclinic	monoclinic	triclinic
Space group	$P\bar{1}$	$P\bar{1}$	$C2/c$	$P\bar{1}$
a/Å	12.4075(10)	16.6237(9)	30.4177(4)	15.136(4)
b/Å	24.3178(11)	21.0590(9)	18.7056(3)	15.488(4)
c/Å	24.9967(14)	22.3417(9)	45.9758(7)	16.4315(7)
α/°	113.416(5)	72.793(4)	90	92.388(14)
β/°	97.062(7)	77.896(4)	104.2310(10)	107.558(14)
γ/°	103.139(6)	77.606(4)	90	116.41(3)
Volume/Å^3	6545.0(8)	7207.0(6)	25356.6(7)	3218.9(13)
Z	4	4	16	2
ρ_{calc} g/cm^3	1.131	1.053	1.129	1.121
μ/mm^{-1}	1.487	0.196	1.677	0.798
F(000)	2400.0	2448	9280	1172.0
Radiation	CuKα (λ = 1.54184)	MoKα (λ = 0.71073)	CuKα (λ = 1.54184)	CuKα (λ = 1.54184)
Reflections collected	41044	42580	53344	19061
Independent reflections	24099 [R_{int} = 0.0626]	25270 [R_{int} = 0.0590]	24852 [R_{int} = 0.0288]	11853 [R_{int} = 0.0357]
Data/restr./ parameters	24099/3/ 1492	25270/2/ 1384	24852/45/ 1488	11853/0/ 724
GoF on F^2	1.058	1.015	1.066	1.020
Final R indexes [I>=2σ (I)]	R_1 = 0.0671, wR_2 = 0.1740	R_1 = 0.0810, wR_2 = 0.1712	R_1 = 0.0768, wR_2 = 0.2052	R_1 = 0.0428, wR_2 = 0.1028
Final R indexes [all data]	R_1 = 0.0906, wR_2 = 0.2075	R_1 = 0.1370, wR_2 = 0.2004	R_1 = 0.0833, wR_2 = 0.2111	R_1 = 0.0636, wR_2 = 0.1164
Largest diff. peak/hole / e Å^{-3}	0.65/-0.91	0.89/-0.64	1.24/-0.43	0.36/-0.49

Table S2. Summary of crystallographic data for polymorphic crystal structure of [V(L^{sq1})(HL^{ox})].

Identification code	V(HL)(L)_SI
Empirical formula	$C_{68}H_{89}N_4O_4V$
Formula weight	1077.37
Temperature/K	120.01(10)
Crystal system	orthorhombic
Space group	Pbca
a/Å	23.2152(3)
b/Å	18.9470(2)
c/Å	58.8131(10)
α/°	90
β/°	90
γ/°	90
Volume/Å^3	25869.4(6)
Z	16
ρ_{calc}g/cm^3	1.106
μ/mm^{-1}	1.644
F(000)	9280.0
Crystal size/mm^3	0.132 × 0.094 × 0.035
Radiation	CuKα (λ = 1.54184)
Reflections collected	57395
Independent reflections	24271 [R_{int} = 0.0608, R_{sigma} = 0.0772]
Data/restraints/parameters	24271/136/1497
Goodness-of-fit on F^2	1.045
Final R indexes [I>=2σ (I)]	R_1 = 0.0634, wR_2 = 0.1302
Final R indexes [all data]	R_1 = 0.1006, wR_2 = 0.1466
Largest diff. peak/hole / e Å^{-3}	0.49/-0.44

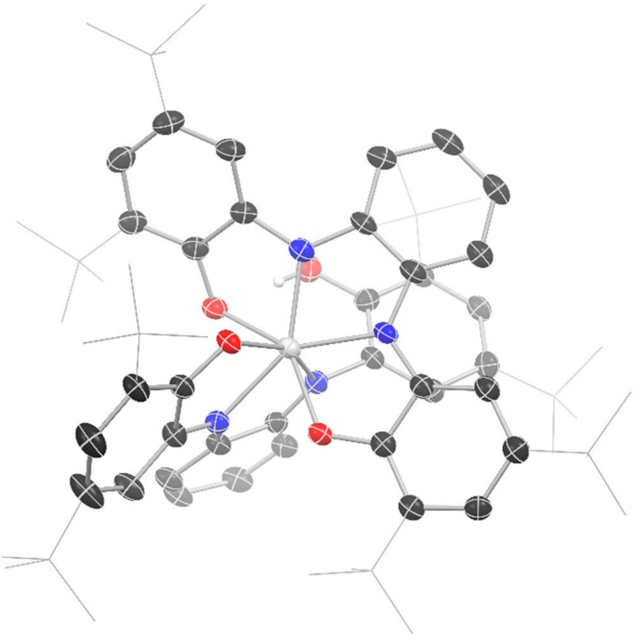

Figure S1. Illustration of molecular structure of orthorhombic polymorph of [V(L^{sq1})(HL^{ox})] measured by single crystal XRD. Only one of the two molecular units present in the asymmetric unit is shown. Non-OH hydrogen atoms are omitted for clarity. Thermal ellipsoids are presented at 50% probability level.

Powder X-ray diffraction analysis: The structural similarity between the bulk powder of [V(L^{sq1})(HL^{ox})] and the two polymorphs obtained by crystallization of [V(L^{sq1})(HL^{ox})], from either acetonitrile or methanol, was studied by powder X-ray diffraction (PXRD, Figure 2). The bulk

material is rather amorphous, but the most significant peaks are clearly visible and correspond to the monoclinic phase. On the other hand, no peaks of the orthorhombic phase are present in the experimental PXRD pattern. The PXRD pattern was measured at 298 K and therefore shifted (0.2°/2θ) to allow better comparison between the simulated patterns which were calculated from the single crystal X-ray structures measured at 175 and 120 K. The powder X-ray diffraction measurements were performed using a Huber G670 detector (Cu-Kα radiation, λ = 1.5406 Å). For each individual measurement, the exposure time was set to 30 min and with a total of 20 scans.

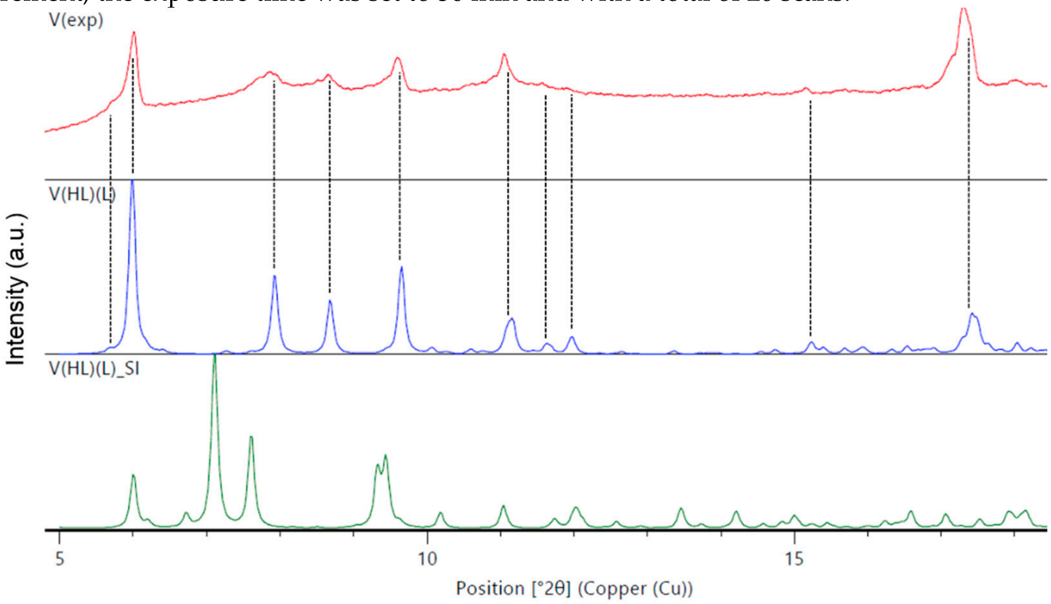

Figure S2. Comparison between the experimental PXRD pattern (top/red) of the bulk material of [V(L^{sq1})(HL^{ox})] and monoclinic (middle/blue) and orthorhombic (bottom/green) polymorphs simulated from single crystal XRD data.

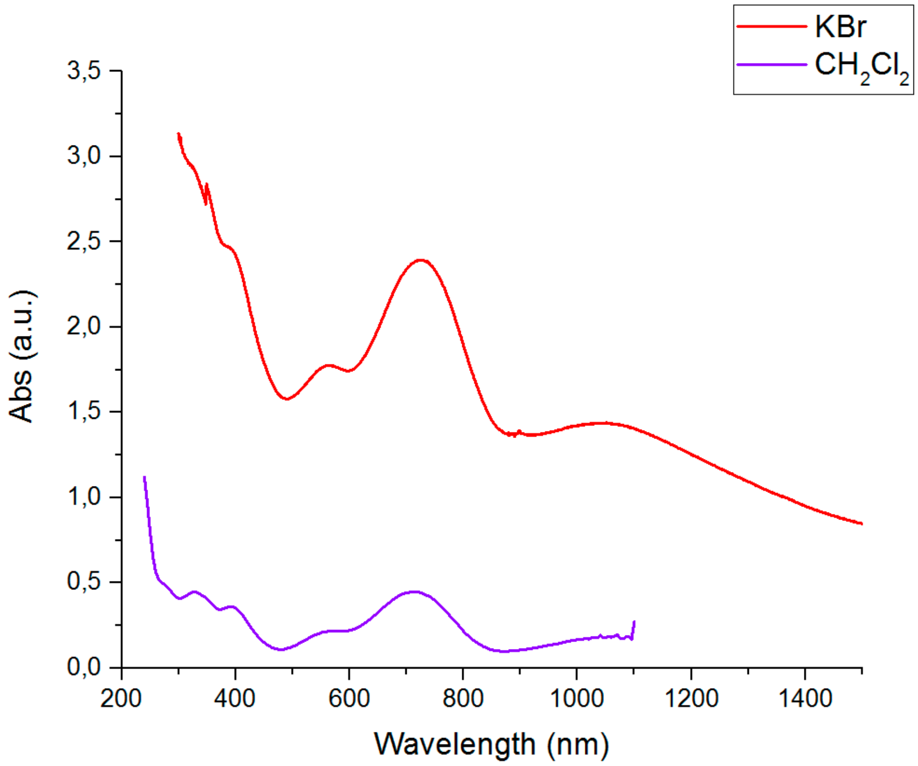

Figure S3. The UV-vis-NIR spectra of [Ti(L^{ox})$_2$].

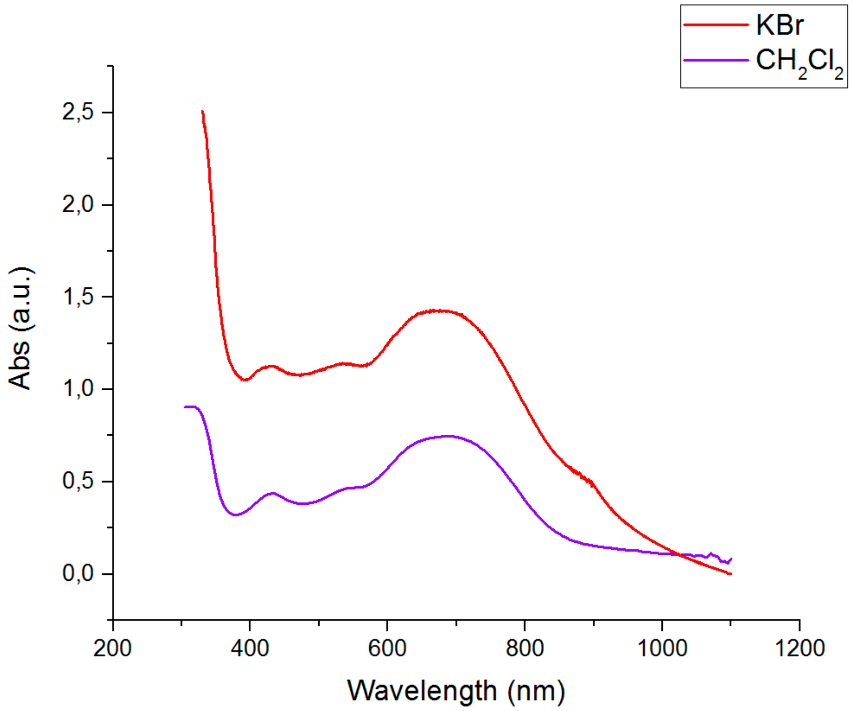

Figure S4. The UV-vis-NIR spectra of [V(L^{sq1})(HL^{ox})].

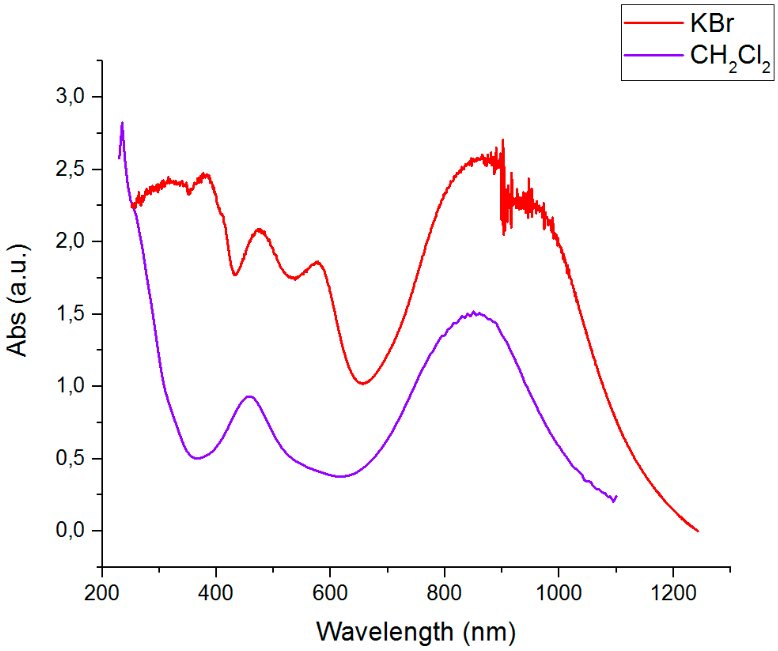

Figure S5. The UV-vis-NIR spectra of [Ni(HL^{ox})$_2$].

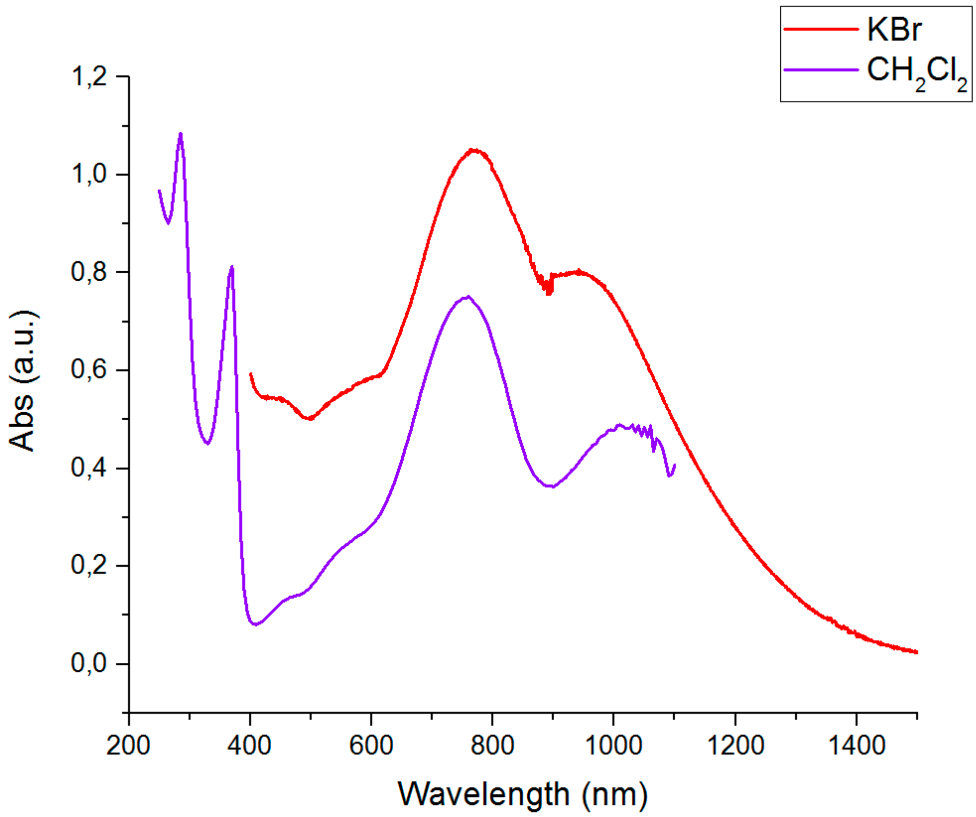

Figure S6. The UV-vis-NIR spectra of [Zr(L^{ox})$_2$].

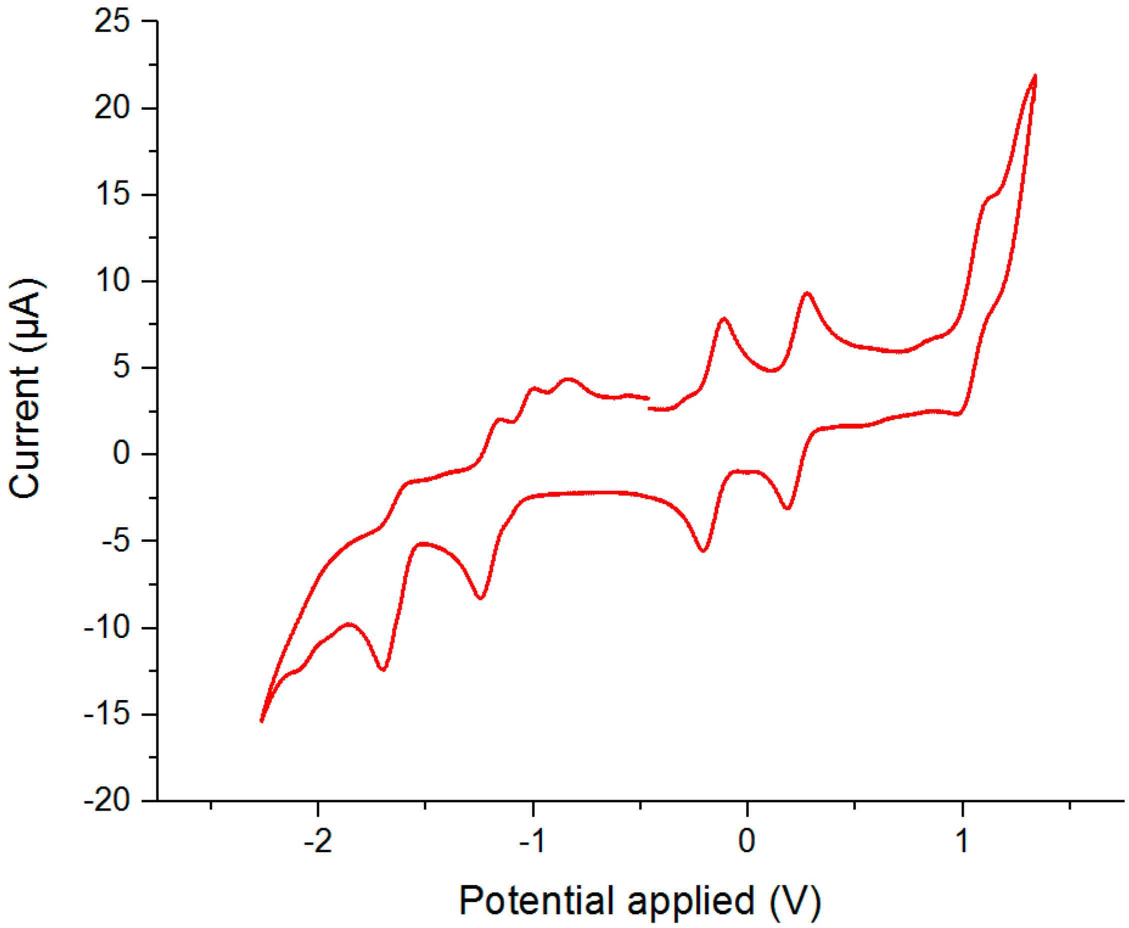

Figure S7. Cyclic voltammogram of [Ti(L^{ox})$_2$]. Cyclic voltammetry is performed at RT vs. Fc/Fc^+, in DCM with 100 mVs^{-1} scan rate and rotation is clockwise.

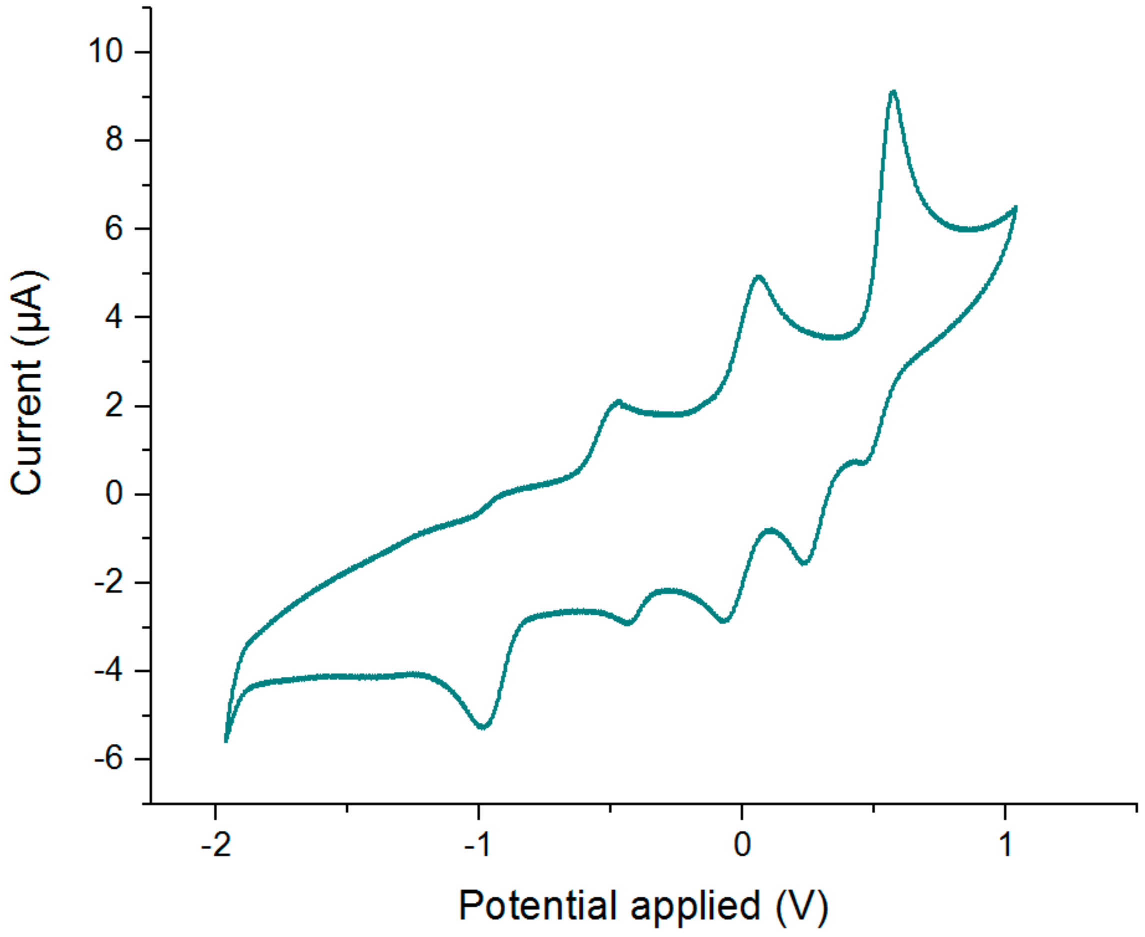

Figure S8. Cyclic voltammogram of [V(L^{sq1})(HL^{ox})]. Cyclic voltammetry is performed at RT vs. Fc/Fc^+, in DCM with 100 mVs^{-1} scan rate and rotation is clockwise.

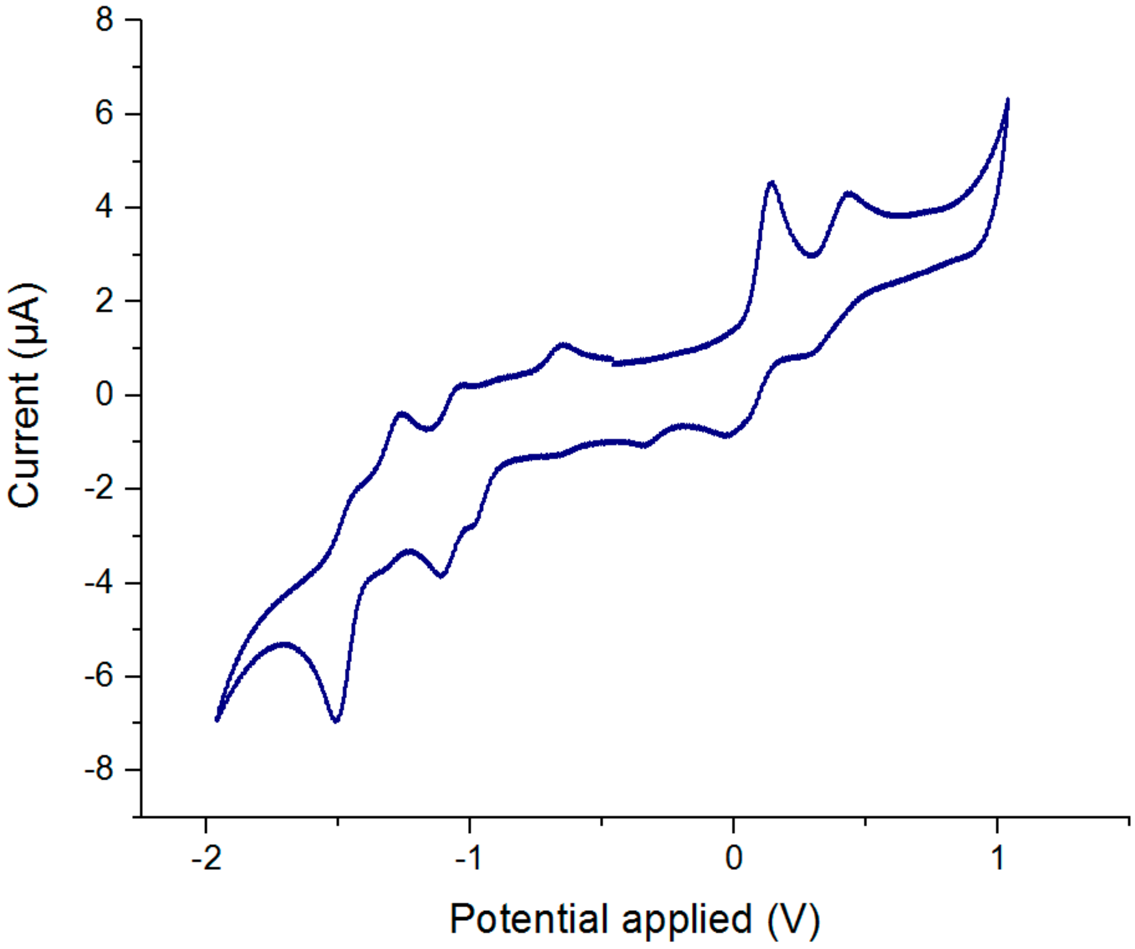

Figure S9. Cyclic voltammogram of [Ni(HL^{ox})$_2$]. Cyclic voltammetry is performed at RT vs. Fc/Fc$^+$, in DCM with 100 mVs^{-1} scan rate and rotation is clockwise.

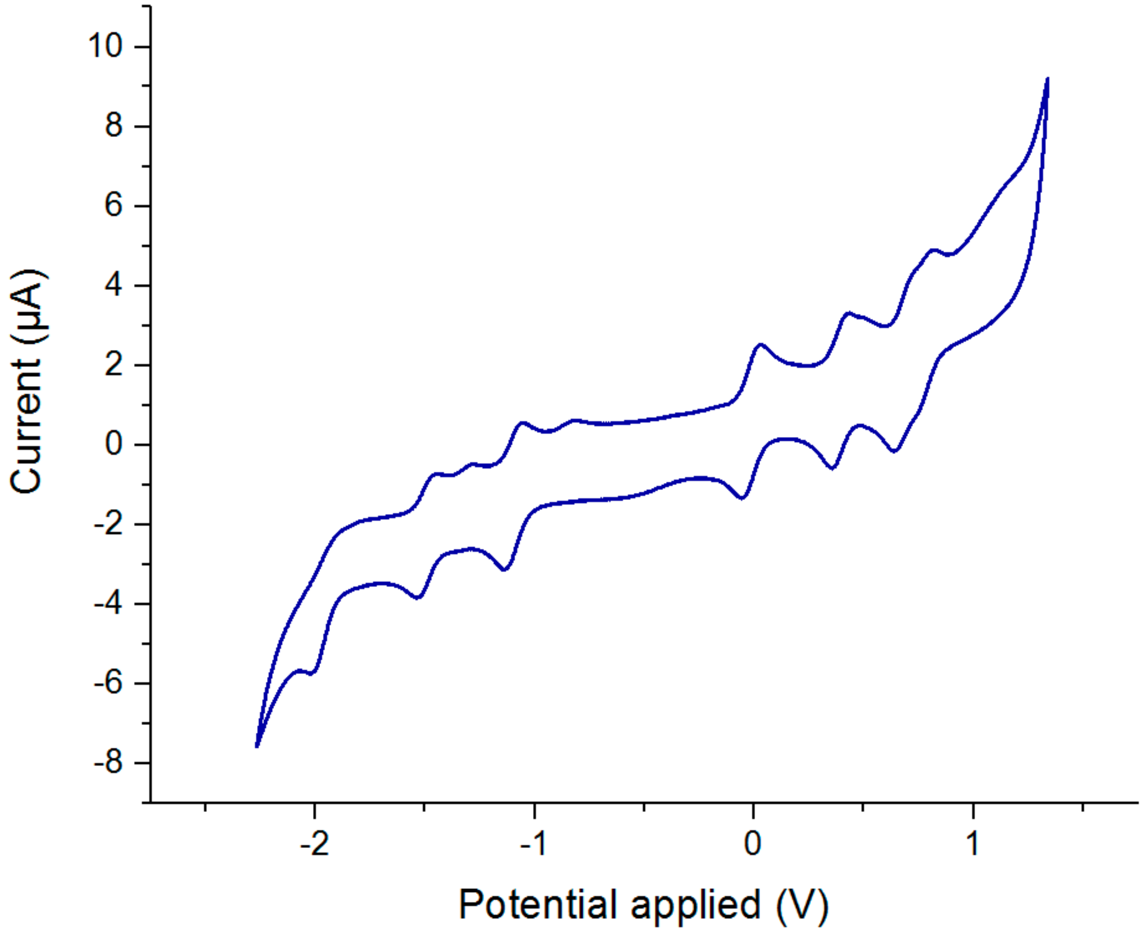

Figure S10. Cyclic voltammogram of [Zr(L^{ox})$_2$]. Cyclic voltammetry is performed at RT vs. Fc/Fc^+, in DCM with 100 mVs^{-1} scan rate and rotation is clockwise.

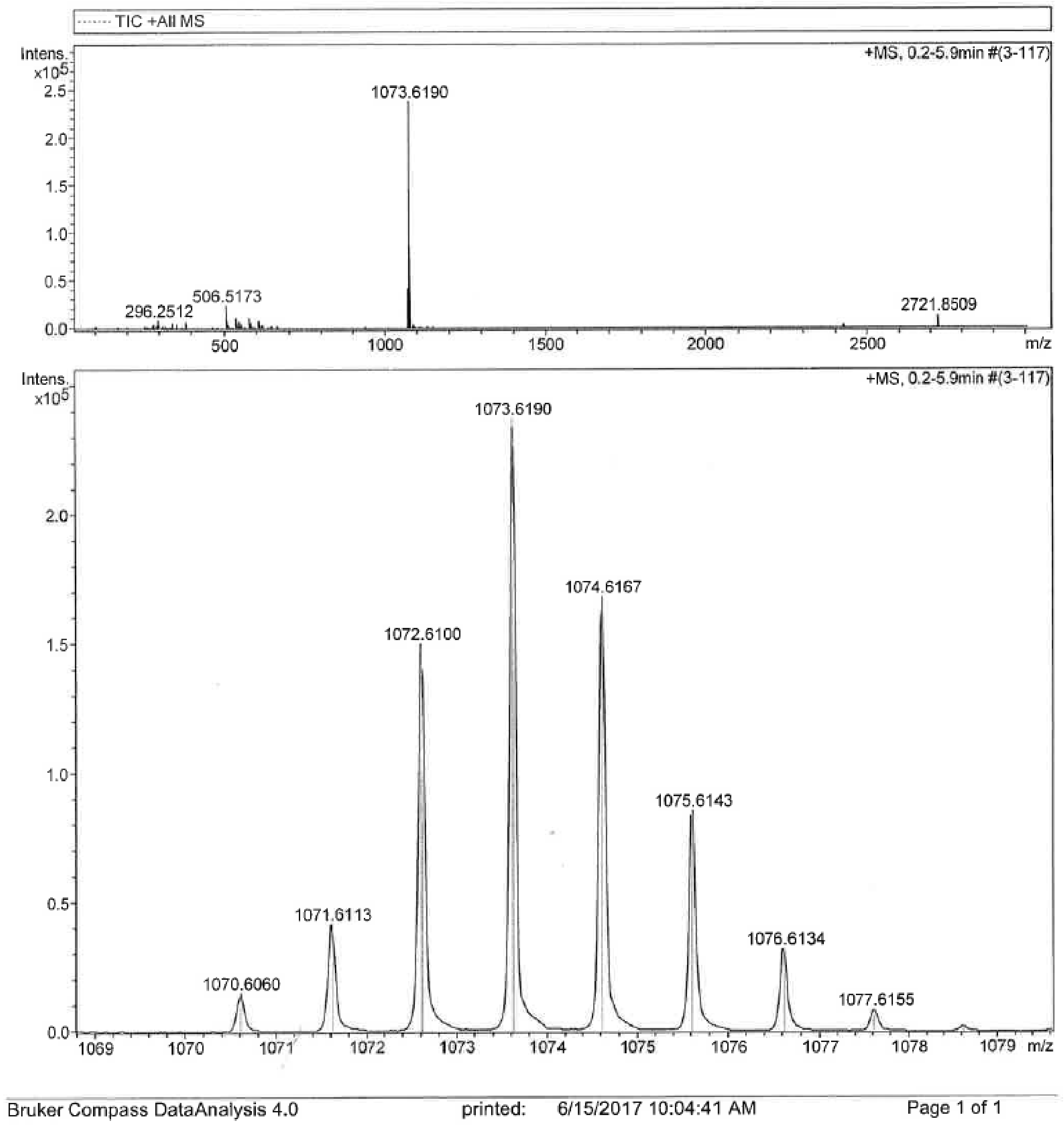

Figure S11. ESI-MS(+) for [Ti(L^{ox})$_2$].

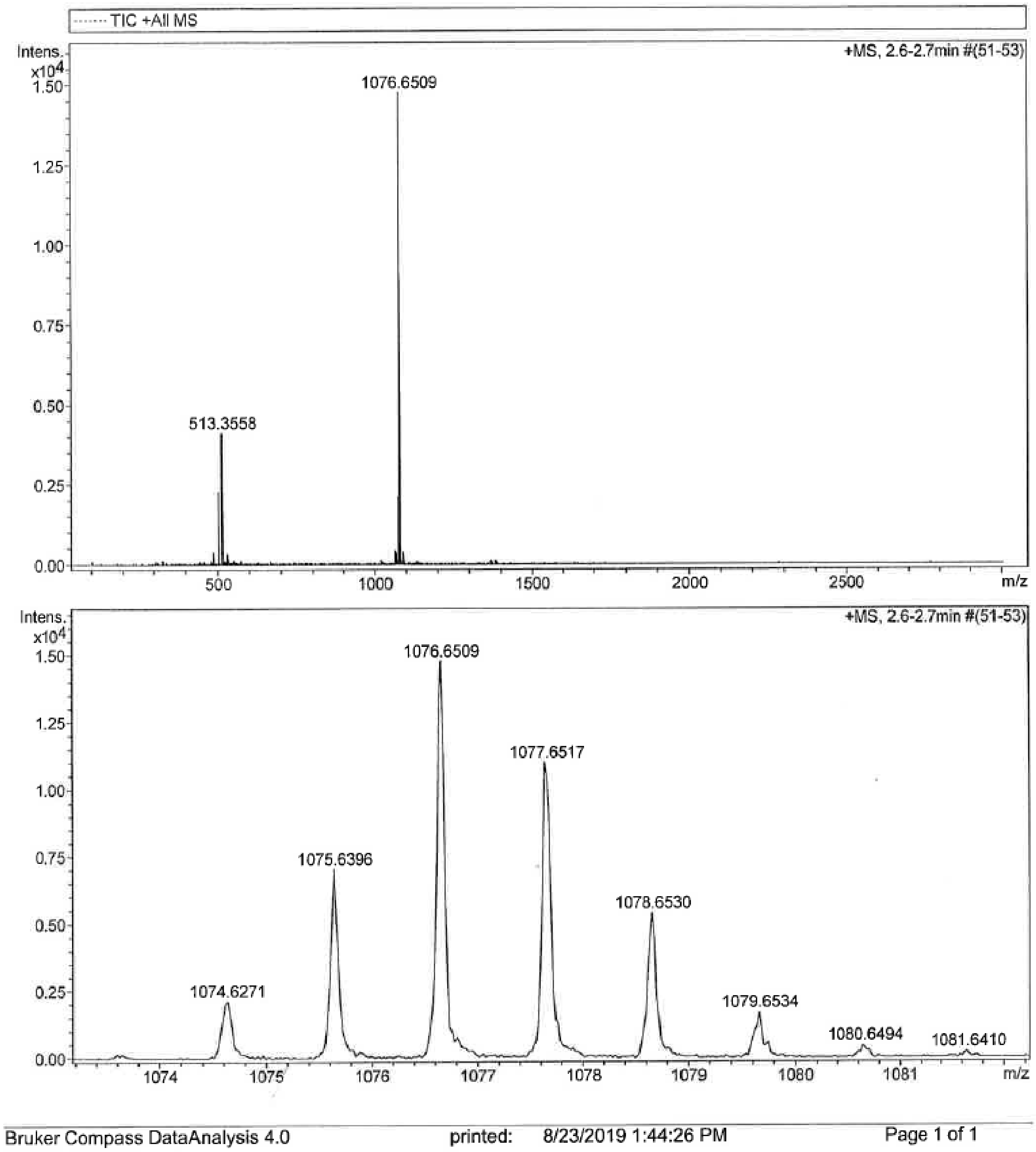

Figure S12. ESI-MS(+) in CH_2Cl_2 for [V(L^{sq1})(HL^{ox})].

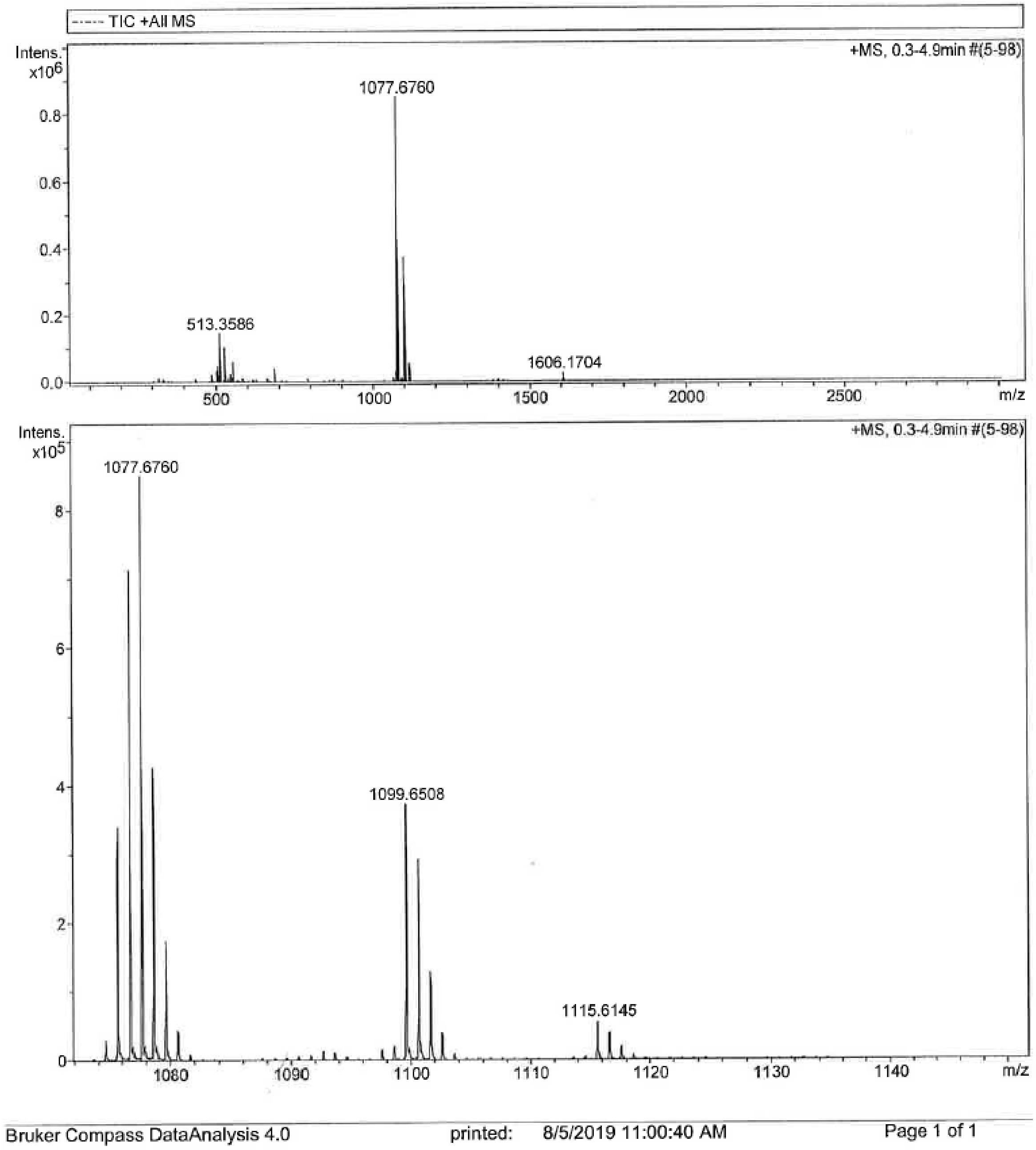

Figure S13. ESI-MS(+) in MeCN for [V(L^{sq1})(HL^{ox})].

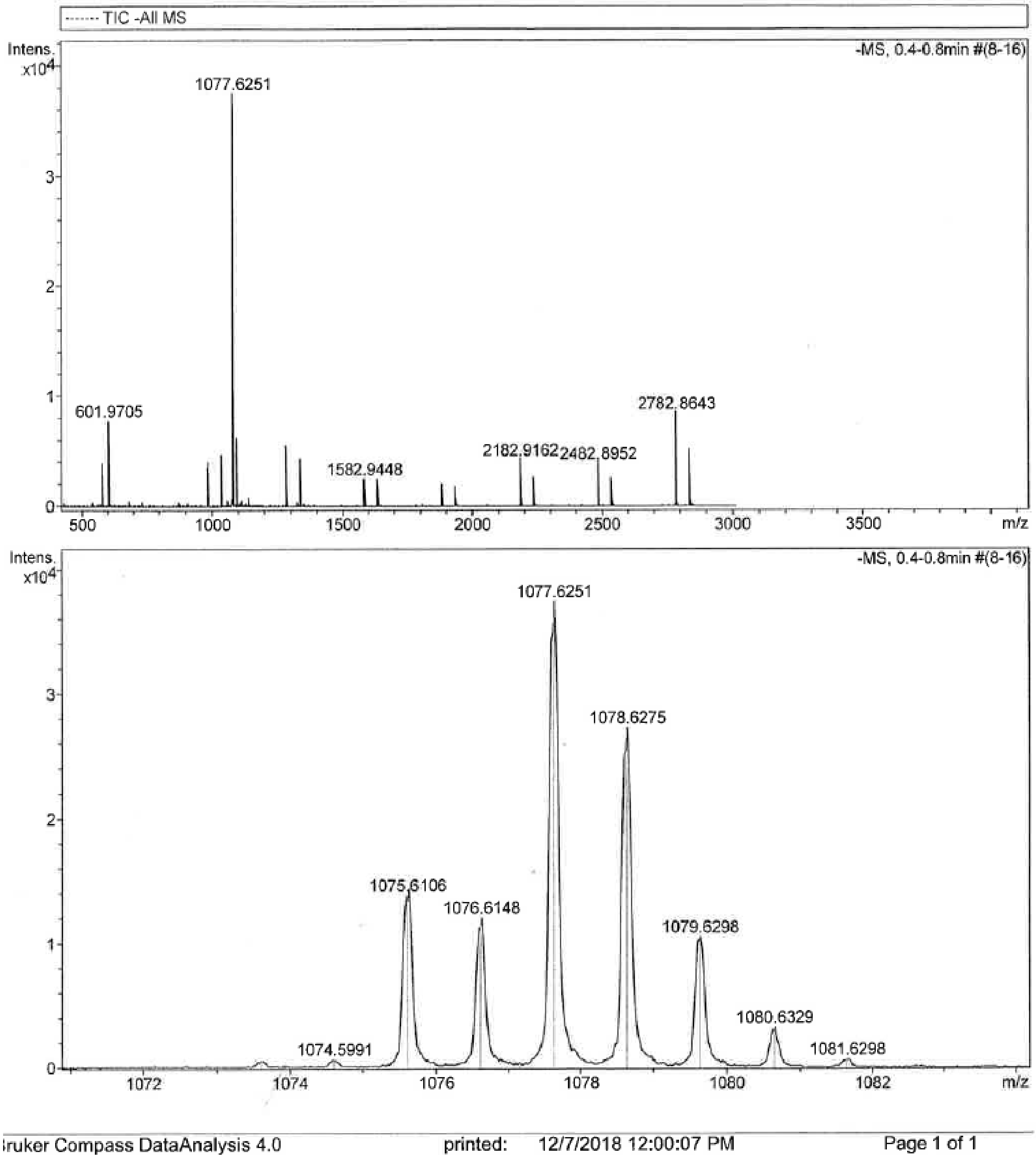

Figure S14. ESI-MS(-) in MeCN for [V(L^{sq1})(HL^{ox})].

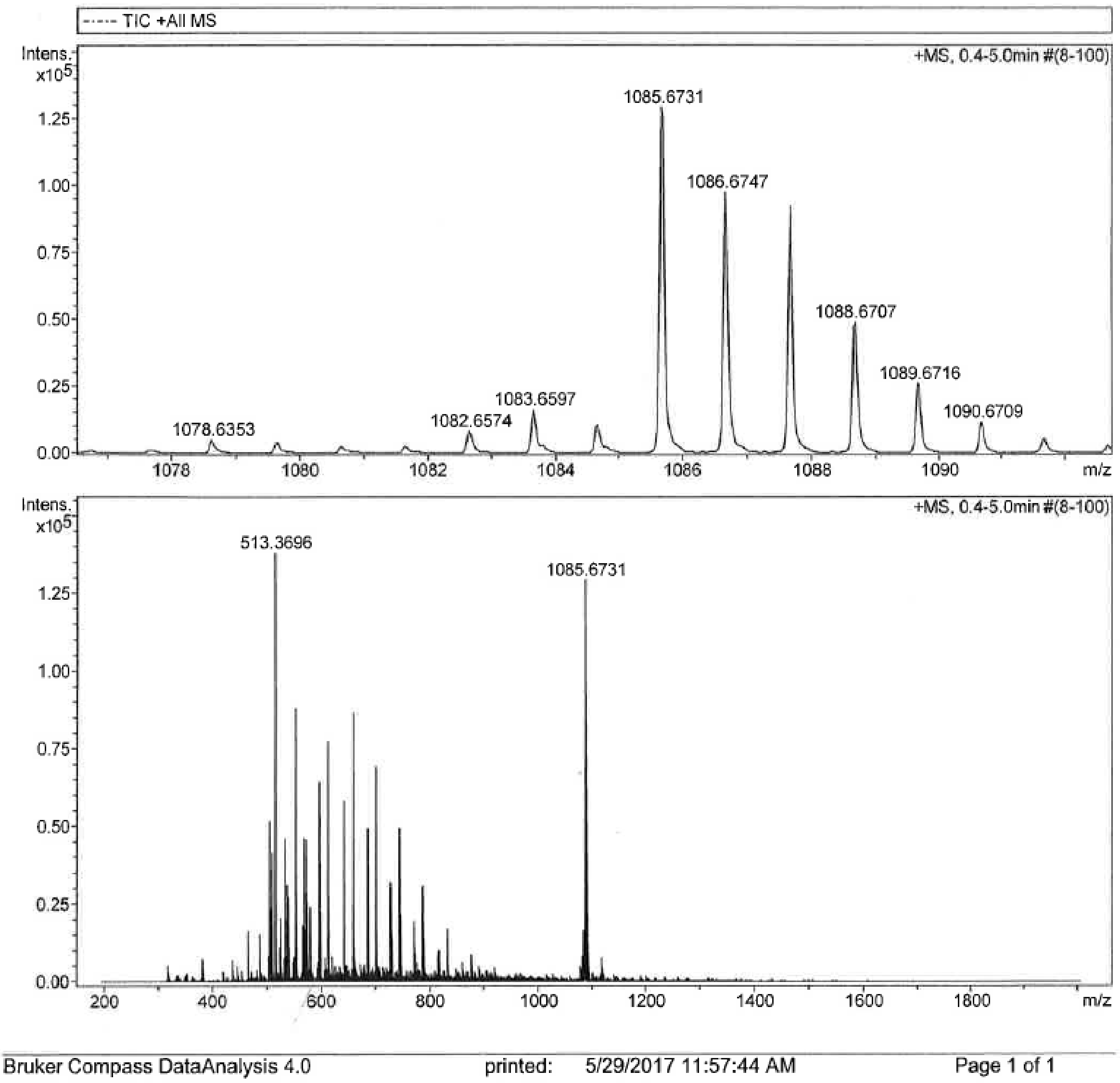

Figure S15. ESI-MS(+) for [Ni(HL^{ox})$_2$].

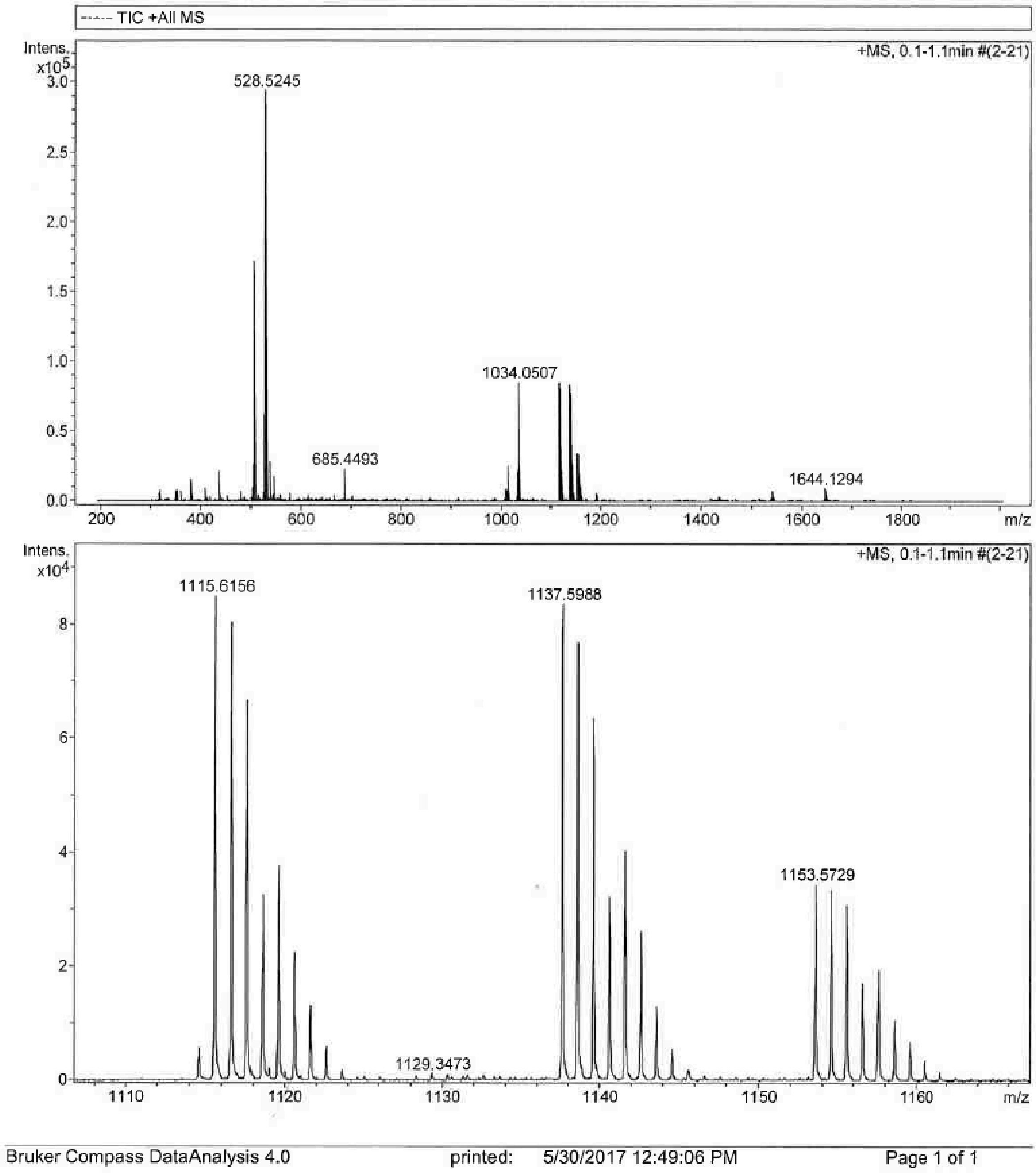

Figure S16. ESI-MS(+) for [Zr(L^{ox})$_2$].

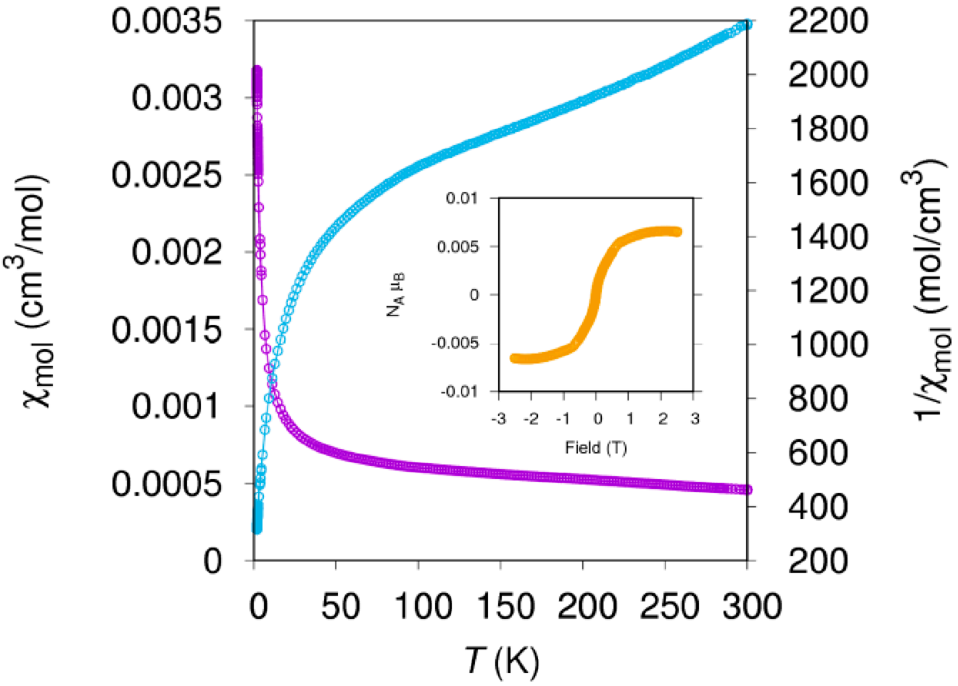

Figure S17. Molar magnetic susceptibility of [V(L^{sq1})(HL^{ox})] in function of temperature (purple) in cgs units. Its reciprocal (blue). The inset displays the field dependence curve at 2 K.

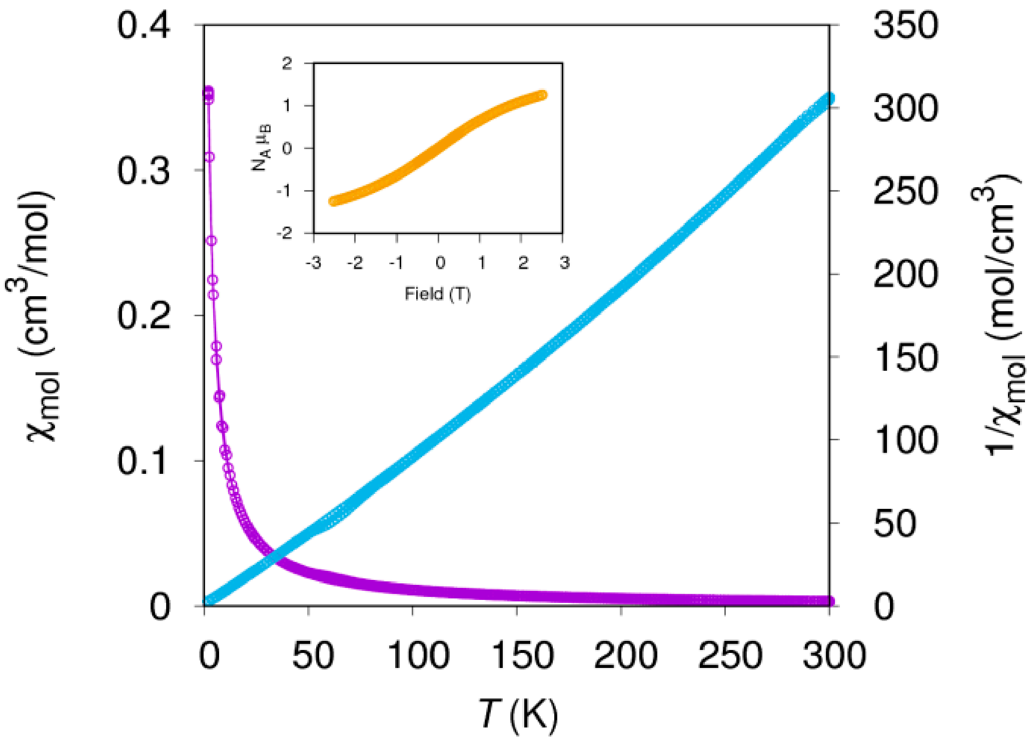

Figure S18. Molar magnetic susceptibility of [Ni(HL^{ox})$_2$] in function of temperature (purple) in cgs units. Its reciprocal (blue). The inset displays the field dependence curve at 2 K.

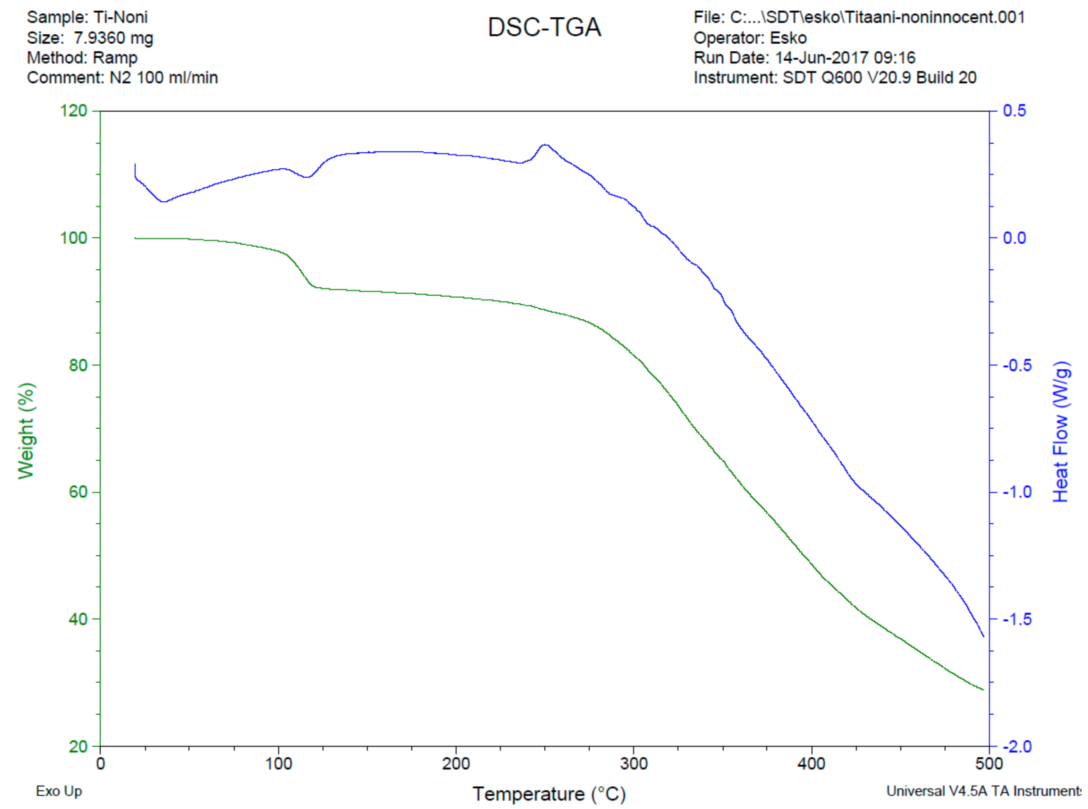

Figure S19. TGA/DSC with the heating rate of 5 K/min for $[Ti(L^{ox})_2]\cdot CH_3CN$ crystals.

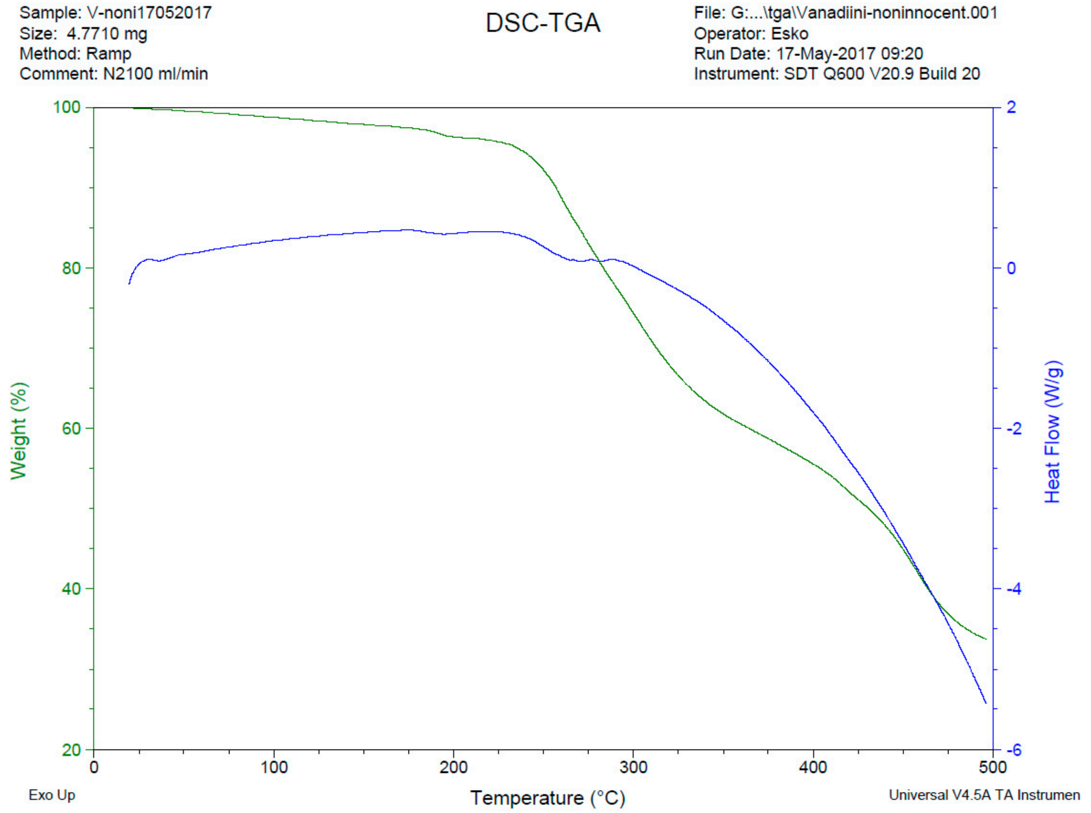

Figure S20. TGA/DSC with the heating rate of 5 K/min for $[V(L^{sq1})(HL^{ox})]$.

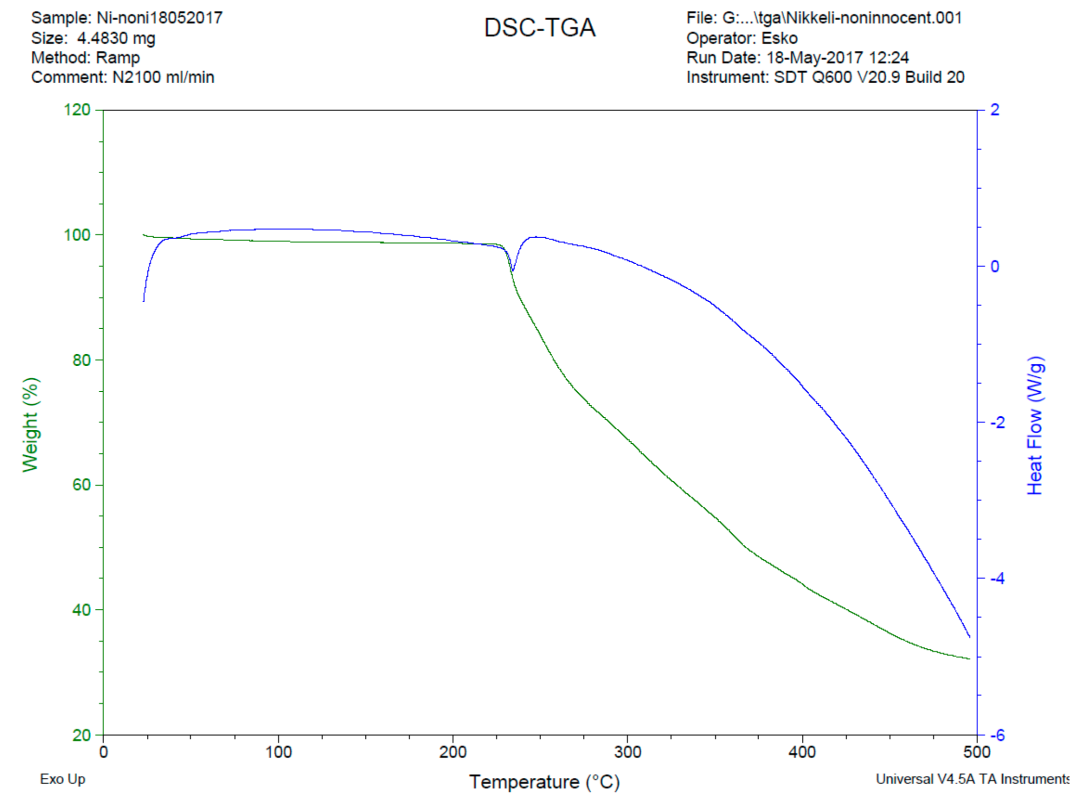

Figure S21. TGA/DSC with the heating rate of 5 K/min for [Ni(HL^{ox})$_2$].

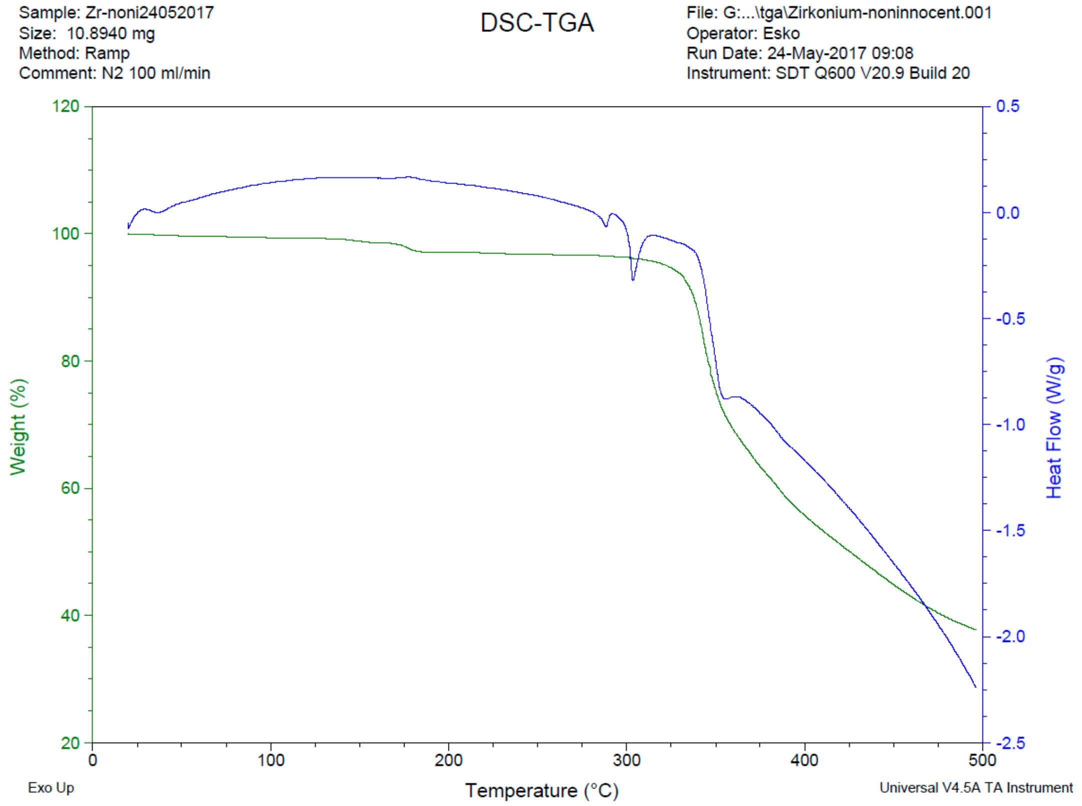

Figure S22. TGA/DSC with the heating rate of 5 K/min for [Zr(L^{ox})$_2$].

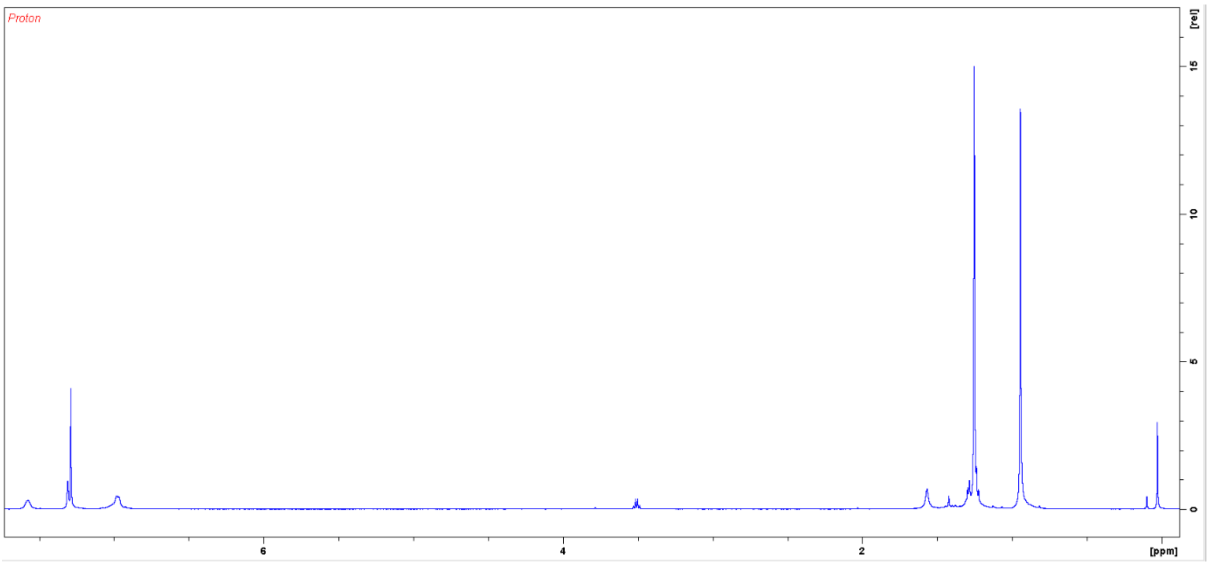

Figure S23. ^{1}H-NMR spectrum for [Ti(L^{ox})$_2$].

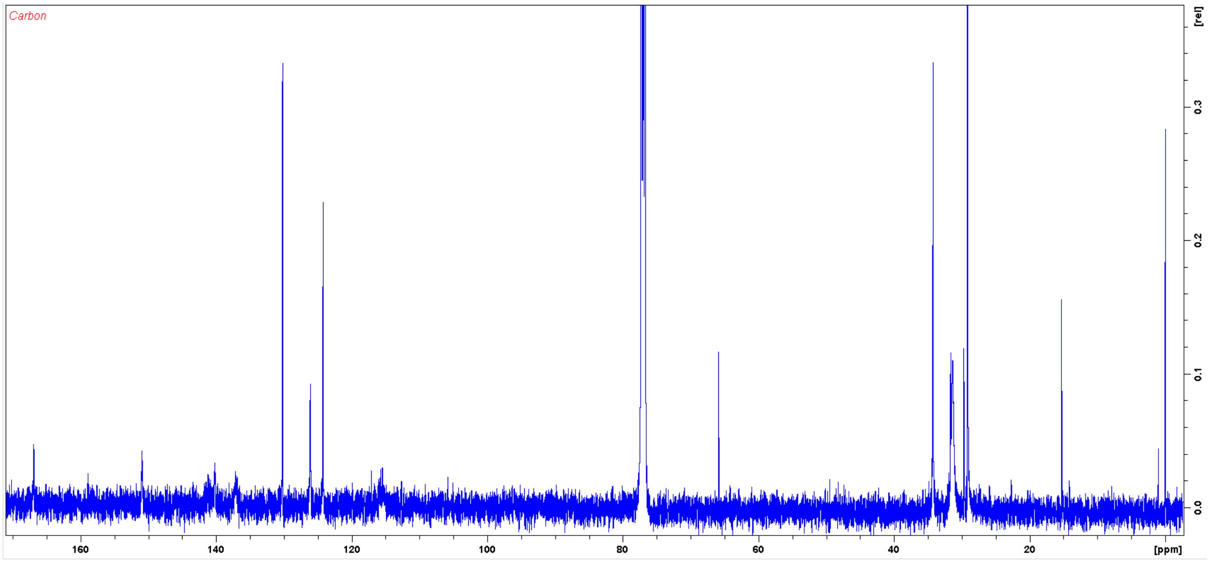

Figure S24. ^{13}C-NMR spectrum for [Ti(L^{ox})$_2$].

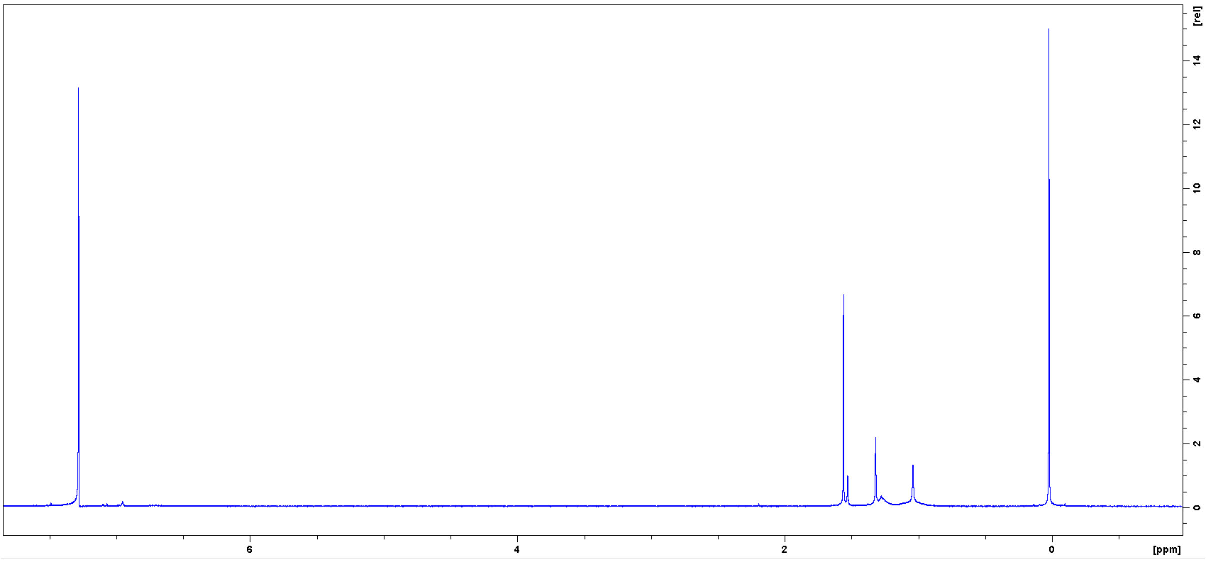

Figure S25. ^{1}H-NMR spectrum for [V(L^{sq1})(HL^{ox})].

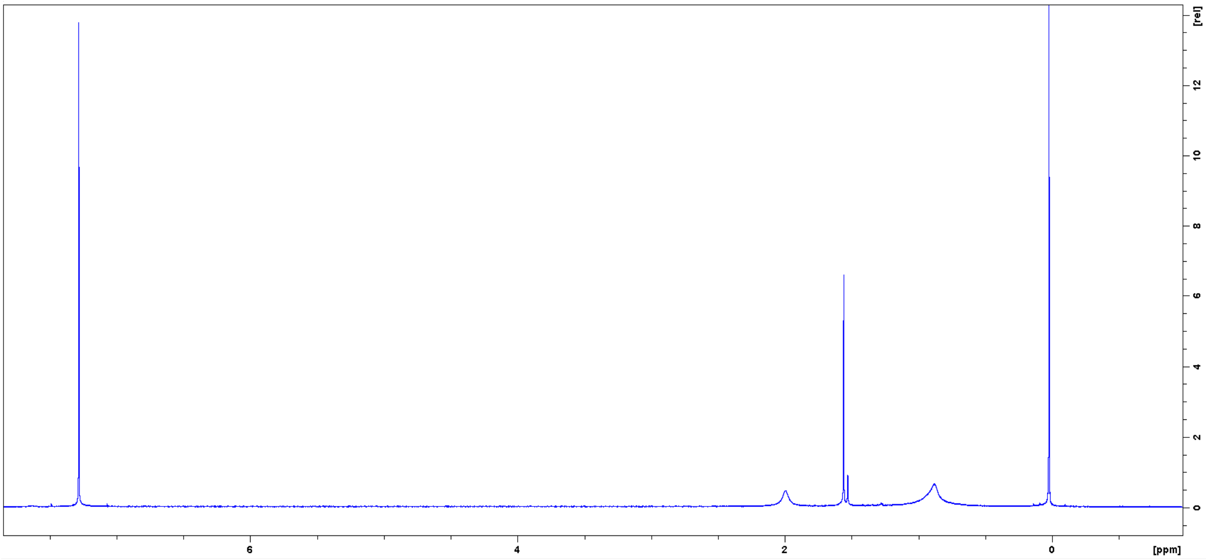

Figure S26. ^{1}H-NMR spectrum for [Ni(HL^{ox})$_2$].

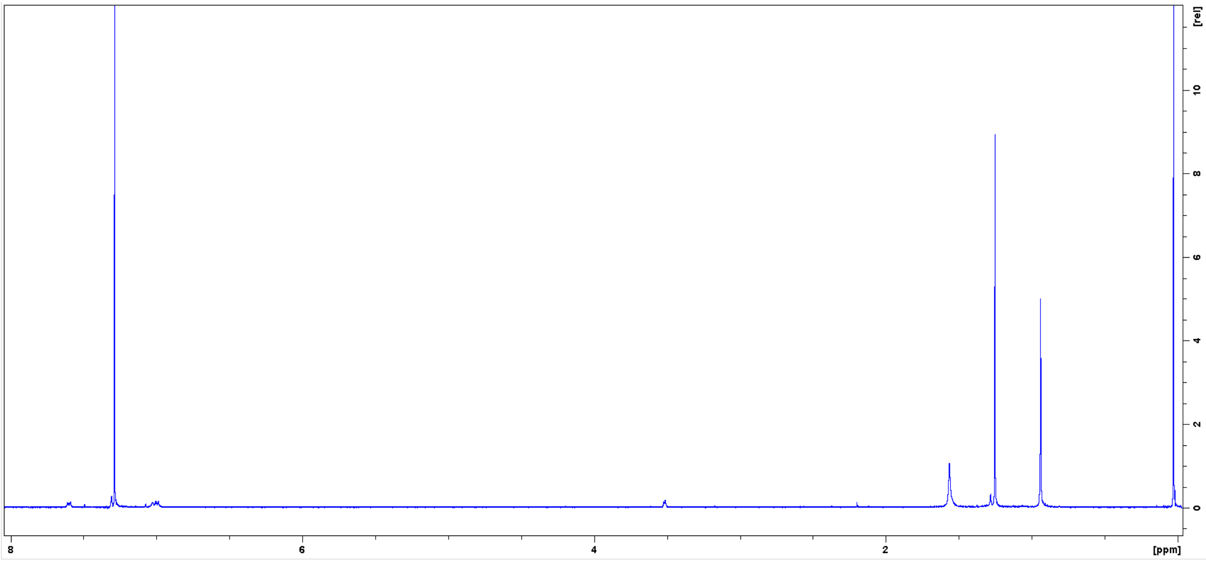

Figure S27. ^{1}H-NMR spectrum for [Zr(L^{ox})$_2$].

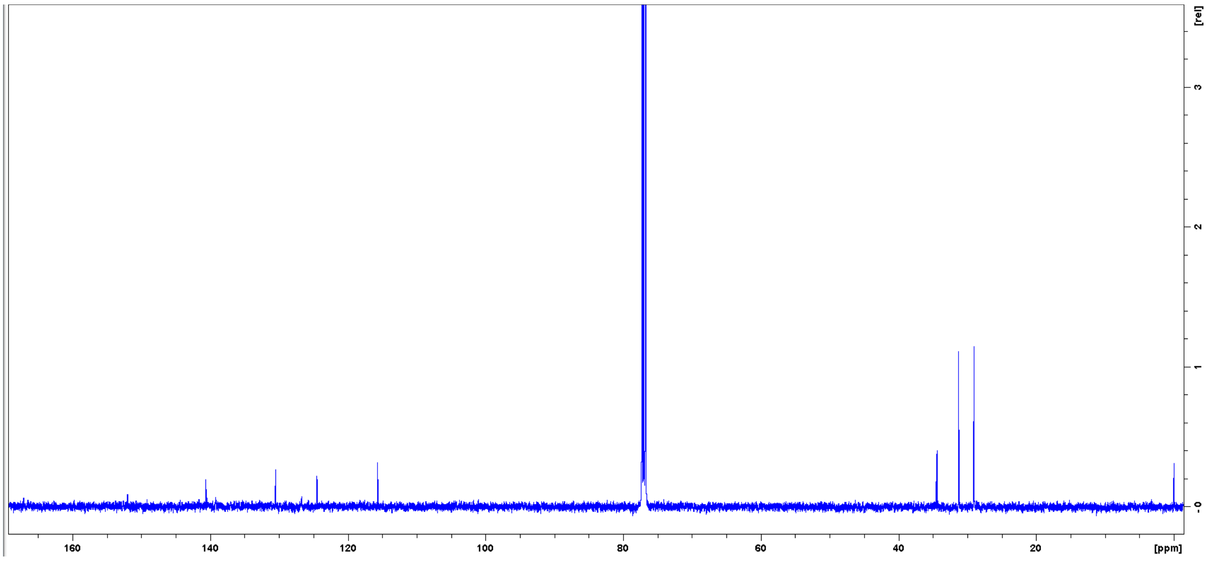

Figure S28. ^{13}C-NMR spectrum for [Zr(L^{ox})$_2$].

Evans' method was carried out as following: samples were dissolved in $CDCl_3$/tBuOH –mixture (95/5 ratio by volume). Sample solutions were then placed in ordinary NMR tubes along with a make-shift ampule of similar, blank $CDCl_3$/tBuOH solution as internal reference. When the concentration of the sample solutions are known, we can then by using the following equation to calculate the effective magnetic moment of the complexes:

$$\mu(eff) = 798 \times \left(\frac{T \times 3\Delta f}{1000 f \times c}\right)^{0.5} (\mu_B) \quad (1)$$

Units are SI units and T is temperature, f is the operating frequency of the nmr apparatus, Δf is the difference of the chemical shifts of the protons and c is concentration in mol/dm^3.

In this case, 5.3 mg of V complex and 3.9 mg of Ni complex were dissolved in 2mLs of $CDCl_3$/tBuOH –solutions. Then ^{1}H-NMR measurements were conducted and from the spectra chemical shifts were calculated. 12.84 Hz for Ni complex and 0.97 Hz for V complex. The sample temperature was 298 K during measurements and NMR apparatus' operating frequency was 500 MHz.

Therefore, by using the equation above, we obtained values for

μ_{eff}(V complex) = 0.66 μ_B and μ_{eff}(Ni complex) = 2.84 μ_B.

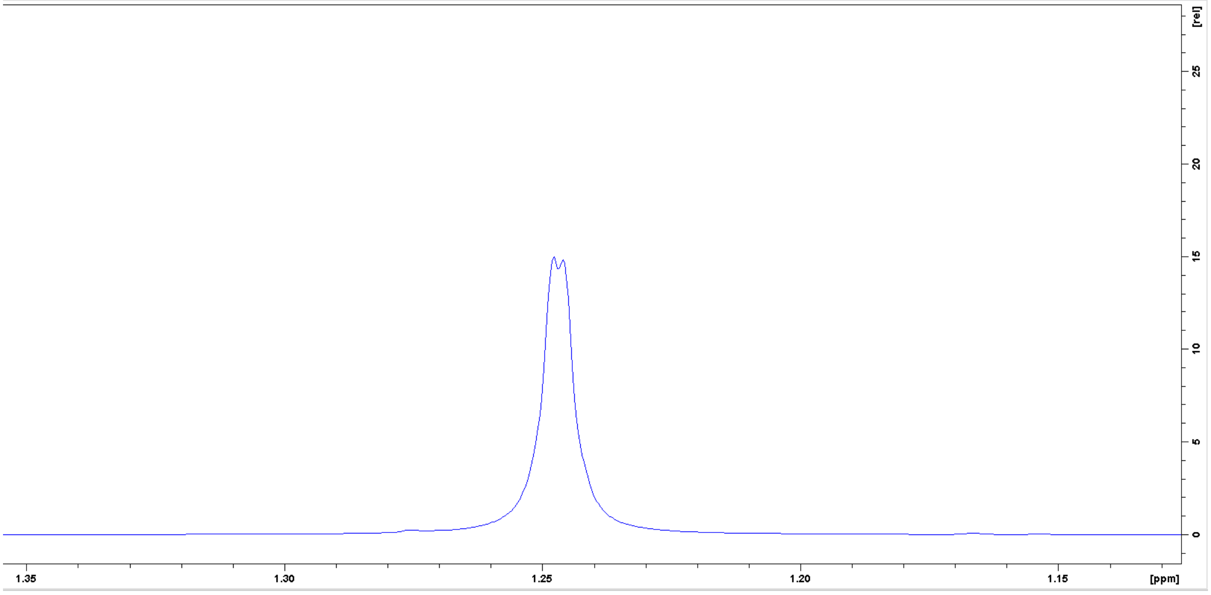

Figure S29. Evans' NMR measurement for [V(L^{sq1})(HL^{ox})].

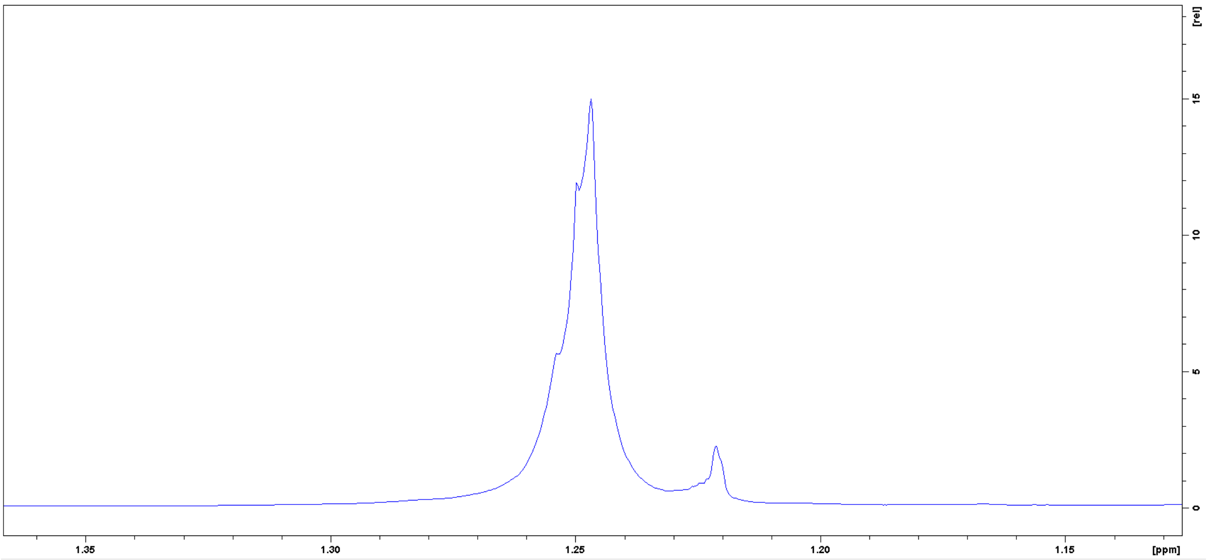

Figure S30. Evans' NMR measurement for [Ni(HL^{ox})$_2$].

Table S3. Optimized bond parameters of the ground state broken symmetry singlet and the two low-lying excited states of the modified structure of [V(L)(HL)].

	XRD	Broken symmetry singlet (S=0)	Triplet (S=1)	Closed shell singlet (S=0)
V1A-O2A	1.971	1.995	2.033	1.969
V1A-O3A	1.950	1.936	1.999	1.941
V1A-O4A	2.054	2.080	2.111	2.054
V1A-N7A	2.071	2.033	2.067	2.024
V1A-N14A	2.155	2.136	2.122	2.168
V1A-N27A	2.052	2.052	2.113	2.015
V1A-N34A	1.989	2.050	2.128	1.960
N7A-C6A	1.425	1.421	1.420	1.423
N7A-C8A	1.322	1.321	1.324	1.321
N14A-C13A	1.323	1.319	1.324	1.318
N14A-C15A	1.362	1.343	1.347	1.342
N27A-C26A	1.397	1.380	1.371	1.385
N27A-C28A	1.392	1.361	1.337	1.363
N34A-C33A	1.371	1.354	1.325	1.363
N34A-C35A	1.402	1.371	1.366	1.389

Table S4. Optimized cartesian coordinates for [(V(L)(HL)] from DFT calculations (in Å).

Broken symmetry singlet (*S* = 0)

V	-0.024195	0.137751	0.171797
O	0.231300	-2.635729	-2.466783
O	1.525430	1.046411	1.040219
O	-1.034676	0.533046	1.775179
O	1.495010	-1.081125	-0.559268
N	-1.138692	-1.491471	-0.313694
N	0.502856	-1.265285	1.693221
N	-1.580577	1.316077	-0.459115
N	0.589724	0.976801	-1.595288
C	-1.778505	-1.664526	-1.570887
C	-1.044520	-2.244878	-2.616765
C	-1.664480	-2.446010	-3.846924
C	-2.981584	-2.068208	-4.038700
H	-3.448748	-2.234669	-5.002786
C	-3.698560	-1.469156	-3.011447
C	-3.093455	-1.269857	-1.780838
H	-3.638409	-0.818372	-0.960703
C	-0.988876	-2.553199	0.458403
C	-1.646050	-3.799693	0.225666
H	-2.330807	-3.875623	-0.608368
C	-1.407956	-4.856135	1.039827
H	-1.913753	-5.796434	0.851021
C	-0.477152	-4.770130	2.114529
H	-0.260556	-5.654536	2.701386
C	0.165459	-3.605713	2.384717
H	0.910513	-3.575704	3.164057
C	-0.079871	-2.444471	1.592731
C	1.352273	-0.733694	2.587955
C	1.677944	-1.214344	3.877652
H	1.156044	-2.067222	4.285528
C	2.599336	-0.558247	4.642500
C	3.238117	0.602841	4.150005
H	3.976968	1.099837	4.768936
C	2.912435	1.132369	2.934472
C	1.918389	0.516812	2.134164
C	-2.275734	0.940039	1.679682
C	-3.175356	0.937079	2.743259
C	-4.483268	1.325251	2.514274
H	-5.197723	1.322838	3.329340
C	-4.894421	1.688125	1.234300
C	-3.997582	1.720804	0.176461
H	-4.364936	1.953417	-0.811742
C	-2.659505	1.381720	0.398839
C	-1.494322	1.971383	-1.649321
C	-2.401095	2.876130	-2.225936
H	-3.269269	3.208414	-1.680701
C	-2.172216	3.367775	-3.494726
H	-2.885659	4.056466	-3.932059
C	-1.050297	2.970139	-4.229284
H	-0.918585	3.311353	-5.249155
C	-0.102053	2.159273	-3.652894
H	0.757816	1.832917	-4.219692
C	-0.273154	1.717958	-2.329448
C	1.889016	0.719178	-1.949968
C	2.750931	1.475727	-2.752172

H 2.435164 2.431642 -
3.147429
C 4.035329 1.023173 -
2.976971
C 4.470831 -0.180206 -
2.416919
H 5.476428 -0.531253 -
2.621489
C 3.645467 -0.921046 -
1.591652
C 2.353544 -0.463475 -
1.331858
H 0.645786 -2.191733 -
1.695660
H 2.840697 -0.924156
5.633061
H -2.836282 0.610459
3.718920
H 4.715700 1.617929 -
3.574266
H 3.982285 -1.841229 -
1.129801
H 3.364910 2.043898
2.564997
H -1.083270 -2.897287 -
4.642349
H -4.723820 -1.155233 -
3.166438
H -5.931661 1.945720
1.053795

Closed shell singlet (*S* = 0)

V -0.029940 0.147270 0.116599
O 0.186713 -2.705062 -2.397010
O 1.479079 1.037039
1.015311
O -1.030421 0.523864 1.736926
O 1.436424 -1.096988 -0.606042
N -1.169826 -1.475138 -0.288776
N 0.511683 -1.261513 1.673274
N -1.577331 1.289402 -0.485594
N 0.583515 0.996859 -1.540273
C -1.804920 -1.658758 -1.548447
C -1.074219 -2.284222 -2.572013
C -1.688804 -2.501656 -3.802621
C -2.997032 -2.105503 -4.014255
H -3.459875 -2.287468 -4.977688
C -3.711241 -1.468289 -3.007957
C -3.112011 -1.247380 -1.778520
H -3.657322 -0.769414 -0.974398
C -1.016257 -2.536631 0.481708
C -1.691221 -3.775874 0.262726
H -2.397553 -3.844441 -0.553677
C -1.439041 -4.835382 1.067805
H -1.956218 -5.771709 0.890673
C -0.477955 -4.759311 2.117466
H -0.250536 -5.647702 2.694101
C 0.177578 -3.600287 2.375933
H 0.943414 -3.578553 3.134961
C -0.081860 -2.435304 1.592569
C 1.375557 -0.738899 2.556687
C 1.732513 -1.220374 3.837583
H 1.237579 -2.085544 4.252430
C 2.655704 -0.548634 4.586484
C 3.262723 0.626644 4.088203
H 4.004204 1.133974
4.695350
C 2.900968 1.158610 2.883472
C 1.911873 0.517929 2.100833
C -2.277350 0.916043 1.649023
C -3.176498 0.906005 2.712271
C -4.487096 1.284793 2.478824
H -5.202215 1.280591 3.293354
C -4.900292 1.641865 1.198348
C -4.002271 1.675454 0.140691
H -4.367592 1.901547 -0.849896
C -2.665047 1.345987 0.369867
C -1.491478 1.980023 -1.657246
C -2.401806 2.880596 -2.235194
H -3.291685 3.185299 -1.708976
C -2.144596 3.401564 -3.485166
H -2.854997 4.088092 -3.930599
C -0.994075 3.035841 -4.190051
H -0.834674 3.402644 -5.197175
C -0.048992 2.224844 -3.604709
H 0.833353 1.929604 -4.153050
C -0.255793 1.756421 -2.299258
C 1.895041 0.730907 -1.912074
C 2.772069 1.511123 -2.662932
H 2.479063 2.490699 -3.015217
C 4.052225 1.040764 -2.905209
C 4.457945 -0.194018 -2.406789
H 5.455705 -0.558984 -2.624605
C 3.609921 -0.954970 -1.618785
C 2.329942 -0.478046 -1.352930
H 0.624053 -2.212575 -1.664163
H 2.924434 -0.915216 5.569711
H -2.836857 0.586776 3.690118
H 4.745561 1.649462 -3.472919
H 3.923435 -1.903104 -1.198544
H 3.327075 2.080860
2.509678
H -1.109303 -2.982722 -4.581573
H -4.730324 -1.142416 -3.178136
H -5.938750 1.893394 1.016889

Triplet (*S* = 1)

V 0.040501 0.125155 0.182216
O 0.143929 -2.065662 -2.745539
O 1.638866 0.994671
1.089920
O -0.937406 0.783437 1.797075
O 1.568648 -1.108683 -0.592412
N -1.121382 -1.513222 -0.304918
N 0.487011 -1.272009 1.715681

N	-1.572301	1.278660	-0.546846
N	0.675783	0.995104	-1.652625
C	-1.872149	-1.635306	-1.504296
C	-1.183480	-1.885213	-2.699922
C	-1.905278	-1.979306	-3.887428
C	-3.281023	-1.834306	-3.890171
H	-3.824134	-1.912432	-4.825139
C	-3.964741	-1.596580	-2.704598
C	-3.255378	-1.497121	-1.517993
H	-3.767416	-1.310402	-0.581013
C	-0.950019	-2.590587	0.444805
C	-1.564636	-3.851594	0.182248
H	-2.241600	-3.933812	-0.657633
C	-1.284514	-4.924010	0.966206
H	-1.749041	-5.878919	0.746689
C	-0.358239	-4.829318	2.039126
H	-0.105486	-5.718711	2.604095
C	0.245569	-3.645958	2.335815
H	0.994926	-3.608101	3.111316
C	-0.046249	-2.476602	1.580052
C	1.322828	-0.762385	2.641640
C	1.590740	-1.259193	3.935902
H	1.036400	-2.106361	4.313617
C	2.484799	-0.620551	4.750907
C	3.153320	0.540332	4.304052
H	3.863285	1.027344	4.963505
C	2.892521	1.079691	3.076466
C	1.937968	0.479615	2.216694
C	-2.183724	1.082503	1.649957
C	-3.096612	1.157173	2.716422
C	-4.416411	1.448283	2.459938
H	-5.125145	1.492196	3.279587
C	-4.866957	1.657412	1.149622
C	-3.985061	1.616380	0.089935
H	-4.366219	1.715580	-0.916260
C	-2.626713	1.358185	0.325871
C	-1.478462	1.851220	-1.751715
C	-2.426457	2.680944	-2.402560
H	-3.328281	2.977326	-1.891455
C	-2.189359	3.136762	-3.669024
H	-2.924136	3.768896	-4.153544
C	-1.010056	2.781773	-4.366600
H	-0.883037	3.091263	-5.397425
C	-0.034262	2.061726	-3.747978
H	0.857174	1.764551	-4.282073
C	-0.206564	1.640166	-2.401387
C	1.980612	0.732125	-1.958349
C	2.855320	1.483109	-2.754358
H	2.533803	2.427577	-3.173893
C	4.151295	1.051347	-2.931727
C	4.586930	-0.135431	-2.329995
H	5.604824	-0.472164	-2.495442
C	3.751815	-0.875437	-1.518855
C	2.435118	-0.450050	-1.308127
H	0.572134	-1.855786	-1.886428
H	2.671841	-0.996642	5.749614
H	-2.737981	0.962067	3.719807
H	4.842184	1.644488	-3.518149
H	4.089748	-1.783004	-1.033536
H	3.370829	1.990022	2.737234
H	-1.355413	-2.175219	-4.800487
H	-5.042359	-1.485238	-2.702535
H	-5.918975	1.838931	0.963821

Pentet ($S = 2$)

V	-0.038133	0.066785	0.256557
O	0.293756	-2.707194	-2.477844
O	1.455948	0.983690	1.057354
O	-1.092311	0.544178	1.869767
O	1.604956	-1.137043	-0.550291
N	-1.076739	-1.498377	-0.312390
N	0.505244	-1.311315	1.689315
N	-1.596843	1.413006	-0.397050
N	0.541402	0.877608	-1.629295
C	-1.681896	-1.660080	-1.585430
C	-0.969410	-2.277549	-2.628039
C	-1.585883	-2.471705	-3.860633
C	-2.889200	-2.054626	-4.066546
H	-3.356237	-2.220027	-5.030965
C	-3.592191	-1.423891	-3.049597
C	-2.985497	-1.234704	-1.817727
H	-3.527881	-0.772673	-1.001373
C	-0.957393	-2.619781	0.461841
C	-1.619397	-3.828135	0.209621
H	-2.297212	-3.893233	-0.631719
C	-1.398345	-4.920054	1.018950
H	-1.913835	-5.849918	0.807636
C	-0.492827	-4.849287	2.083750
H	-0.291343	-5.729261	2.682826
C	0.169508	-3.674917	2.359132
H	0.904322	-3.655504	3.148455
C	-0.065788	-2.526656	1.579266
C	1.349992	-0.782450	2.607825
C	1.712809	-1.285001	3.868675
H	1.252389	-2.182244	4.252949
C	2.613744	-0.599216	4.651784
C	3.177013	0.601654	4.205035
H	3.889810	1.124567	4.832408
C	2.807093	1.144428	2.993664
C	1.873175	0.483750	2.189153
C	-2.279357	1.004548	1.742630
C	-3.196394	1.070504	2.807419
C	-4.477592	1.488175	2.557649
H	-5.202606	1.516024	3.362946
C	-4.882643	1.842073	1.253187
C	-3.995050	1.845875	0.207837
H	-4.349585	2.069309	-0.786731
C	-2.649975	1.470898	0.435044
C	-1.513434	1.996418	-1.638140
C	-2.396224	2.916606	-2.215056
H	-3.214027	3.323172	-1.641690
C	-2.205137	3.345105	-3.517644

H	-2.900490	4.051076	-3.954918
C	-1.139482	2.856741	-4.265553
H	-1.020900	3.150865	-5.301839
C	-0.208490	2.014131	-3.686417
H	0.609890	1.623260	-4.274266
C	-0.340027	1.638353	-2.347233
C	1.829771	0.689413	-1.962705
C	2.653566	1.473526	-2.796882
H	2.281047	2.400339	-3.211267
C	3.949607	1.088122	-3.021874
C	4.471989	-0.089616	-2.445323
H	5.494506	-0.377566	-2.662964
C	3.710159	-0.860977	-1.606971
C	2.384123	-0.477662	-1.322017
H	0.680938	-2.335351	-1.662673
H	2.889911	-0.996725	5.621396
H	-2.873718	0.745941	3.788668
H	4.592945	1.710002	-3.633007
H	4.102298	-1.751784	-1.132147
H	3.207831	2.088698	
2.646143			
H	-1.015470	-2.953672	-4.645929
H	-4.608486	-1.084605	-3.211446
H	-5.918225	2.104664	1.072107
